# A comparison of two informative SNP-based strategies for typing *Pseudomonas aeruginosa* isolates from patients with cystic fibrosis

**DOI:** 10.1186/1471-2334-14-307

**Published:** 2014-06-05

**Authors:** Melanie W Syrmis, Timothy J Kidd, Ralf J Moser, Kay A Ramsay, Kristen M Gibson, Snehal Anuj, Scott C Bell, Claire E Wainwright, Keith Grimwood, Michael Nissen, Theo P Sloots, David M Whiley

**Affiliations:** 1 Queensland Children’s Medical Research Institute, The University of Queensland, Brisbane, Queensland 4029, Australia; 2 Queensland Paediatric Infectious Disease Laboratory, Block 28, Royal Children’s Hospital, Herston Road, Herston, Brisbane 4029, Queensland, Australia; 3 Sequenom Inc., Sequenom Asia Pacific, Brisbane, Queensland 4029, Australia; 4 Department of Thoracic Medicine, The Prince Charles Hospital, Brisbane, Queensland 4032, Australia; 5 Queensland Children’s Respiratory Centre, Royal Children’s Hospital, Brisbane, Queensland 4029, Australia; 6 Microbiology Division, Pathology Queensland Central Laboratory, Brisbane, Queensland 4029, Australia

**Keywords:** *Pseudomonas aeruginosa*, Typing, Cystic fibrosis, MLST, SNP

## Abstract

**Background:**

Molecular typing is integral for identifying *Pseudomonas aeruginosa* strains that may be shared between patients with cystic fibrosis (CF). We conducted a side-by-side comparison of two *P. aeruginosa* genotyping methods utilising informative-single nucleotide polymorphism (SNP) methods; one targeting 10 *P. aeruginosa* SNPs and using real-time polymerase chain reaction technology (HRM10SNP) and the other targeting 20 SNPs and based on the Sequenom MassARRAY platform (iPLEX20SNP).

**Methods:**

An *in-silico* analysis of the 20 SNPs used for the iPLEX20SNP method was initially conducted using sequence type (ST) data on the *P. aeruginosa* PubMLST website. A total of 506 clinical isolates collected from patients attending 11 CF centres throughout Australia were then tested by both the HRM10SNP and iPLEX20SNP assays. Type-ability and discriminatory power of the methods, as well as their ability to identify commonly shared *P. aeruginosa* strains, were compared.

**Results:**

The *in-silico* analyses showed that the 1401 STs available on the PubMLST website could be divided into 927 different 20-SNP profiles (*D*-value = 0.999), and that most STs of national or international importance in CF could be distinguished either individually or as belonging to closely related single- or double-locus variant groups. When applied to the 506 clinical isolates, the iPLEX20SNP provided better discrimination over the HRM10SNP method with 147 different 20-SNP and 92 different 10-SNP profiles observed, respectively. For detecting the three most commonly shared Australian *P. aeruginosa* strains AUST-01, AUST-02 and AUST-06, the two methods were in agreement for 80/81 (98.8%), 48/49 (97.8%) and 11/12 (91.7%) isolates, respectively.

**Conclusions:**

The iPLEX20SNP is a superior new method for broader SNP-based MLST-style investigations of *P. aeruginosa.* However, because of convenience and availability, the HRM10SNP method remains better suited for clinical microbiology laboratories that only utilise real-time PCR technology and where the main interest is detection of the most highly-prevalent *P. aeruginosa* CF strains within Australian clinics.

## Background

Cystic fibrosis (CF) is the most common, lethal autosomal recessive disease in Caucasian populations [1]. Most CF patients die in their third or fourth decade from complications of chronic pulmonary infection. *Pseudomonas aeruginosa* is the predominant pathogen and once it is established within the lungs of CF patients it is rarely eradicated, resulting in increased treatment requirements and an accelerated decline in lung function, quality of life and survival [2]. While many CF patients acquire *P. aeruginosa* from their natural environment, there is also evidence of person-to-person transmission occurring [3]. Delaying or even preventing *P. aeruginosa* infection is an important management goal. Consequently, determining *P. aeruginosa* acquisition pathways and conducting longitudinal surveillance using molecular-based typing techniques are critical steps for developing novel interventions and evidence-based infection control policies to interrupt the spread of transmissible strains within the CF community [4-6].

Recently, multi-locus sequence typing (MLST) has emerged as an important epidemiological tool for investigating temporally and geographically diverse bacteria [7]. It offers a standardised, reproducible and portable typing approach that allows reliable data comparisons by way of a publically accessible web-based database [7,8]. However, when applied to large-scale investigations involving many hundreds or thousands of isolates it is limited by cost and complexity [9]. To circumvent these problems, some researchers have utilised defined sets of informative single nucleotide polymorphisms (SNPs) derived from MLST data to infer genetic relationships between isolates. In essence, it is a narrowed MLST approach and has been applied to various organisms, including pathogens relevant to CF, such as methicillin-resistant *Staphylococcus aureus* and *P. aeruginosa*[10-13]. Selection of appropriate SNPs, including SNP location and total numbers, is an integral facet of informative SNP strategy to ensure a discriminatory, yet cost-effective, typing scheme. However, once an informative SNP approach tailored to a particular purpose is implemented, it will theoretically have limitations in terms of discriminatory power if used beyond its original objectives.

Previously, we have shown that SYBR Green-based real-time polymerase chain reaction (PCR) assays and high-resolution melting (HRM) curve analysis targeting 10 key SNPs in five housekeeping genes (HRM10SNP) can detect the major *P. aeruginosa* strains shared by CF patients in Queensland, Australia [10]. Furthermore, we demonstrated recently that this form of typing can be adapted to the iPLEX MassARRAY platform to allow high-throughput genotyping [14]. However, based on the high levels of genetic diversity observed amongst shared *P. aeruginosa* strains in the national Australian CF study [15] and also internationally amongst patients attending CF clinics [16], we sought to reassess the HRM10SNP and investigate alternative SNP-based typing strategies for identifying a broader range of *P. aeruginosa* strains.

## Methods

### Clinical isolates

To ensure representative and geographical diversity, 506 clinical isolates were sourced randomly from a biobank of CF isolates collected as part of an ongoing national study of shared *P. aeruginosa* strains involving patients attending 11 CF clinics in Australia’s five largest cities [15] (Additional file 1: Table S1). Isolates were incubated on horse blood agar plates for 24-hours at 37°C. Once purity was confirmed, heat-denatured suspensions of each isolate were prepared as described previously [10].

### HRM10SNPAssay

The HRM10SNP assay was performed for each isolate as described previously [10]. Briefly, each heat-denatured isolate was tested using 10 individual PCR reactions using the qPCR SuperMix-UDG (Invitrogen Australia, Mulgrave, NSW, Australia) on the Rotorgene-6000 (Qiagen, Doncaster, Victoria, Australia). Results from each reaction were compiled to provide a 10-SNP profile for each isolate. As reported previously, isolates with 10-SNP profiles of CTCCTCGGCA, TCTTTCGGTA and CCTCCTGATG were determined to be AUST-01, AUST-02 and AUST-06, respectively [10].

### 20-SNP iPLEXMassARRAY(iPLEX20SNP)

The iPLEX20SNP assay was based on the Sequenom MassARRAY platform (Sequenom, Brisbane, Queensland, Australia) and was a modification of a method described previously [14]. Here, SNPs were derived by analysing sequence data on the *P. aeruginosa* PubMLST website [17]. Briefly, 1070 concatenated sequences of *P. aeruginosa* housekeeping genes (*acsA*, *aroE*, *guaA*, *mutL*, *nuoD*, *ppsA*, and *trpE*) were downloaded (12 January, 2012) and investigated for informative SNPs with the aid of the Minimum SNPs software version 2043 [18] and by manual sorting (using BioEdit version 7.0.9.0). Overall, 20 SNPs were identified and SNP positions based on the 2882 bp concatenated *P. aeruginosa* MLST sequence are listed in Tables 1 and 2. Of these 20 SNPs, four were identical to SNPs used in the HRM10SNP assay; SNPs at sites 7, 322, 1152 and 2551 of the iPLEX20SNP assay (Tables 1 and 2) overlapped with SNPs 1, 2, 5 and 10 from the HRM10SNP assay.

**Table 1:** Primers for primary PCR reaction for the iPLEX20SNP

**SNP site**	**Gene target**	**Primers (5′-3′)**
7 and 45	*acsA*	P1 10mer-ACCTTGTGCTTGTCGATGAT
		P2 10mer-GCCACACCTACATCGTCTAT
322, 381 and 387	*acsA*	P1 10mer-ATCAGGTTGCCGAGGTTGTC
		P2 10mer-AGACCGGCGCCTGCCTGATG
416 and 488	*aroE*	P1 10mer-TCGGTGTTGTCGCCGCGCAG
		P2 10mer-CAATGTCACCGTGCCGTTCA
881	*aroE*	P1 10mer-CAGAGGAAGAATGCCTCGG
		P2 10mer-CGACATGATGTATGCCAAGG
894 and 937	*guaA*	P1 10mer-AACATCGTCGACGACGCCAT
		P2 10mer-AACACGCAGGTCAGTTGGTC
1086 and 1152	*guaA*	P1 10mer-GGCGAGGAACTTCACGTCCTG
		P2 10mer-ATGGGCGTGAAGGTGATCCG
1297	*mutL*	P1 10mer-AGAAGACCGAGTTCGACCAT
		P2 10mer-AAGATGGTCTTGCCGTTGTG
1465	*mutL*	P1 10mer-ACCAGCTTGTCGCGCACCAT
		P2 10mer-AGCGCAACGGCCTGCACCT
1865 and 1958	*nuoD*	P1 10mer-TACAGCAGGTGGTTCAGGAT
		P2 10mer-AAGATGGCCGAGCGCCAGT
2169 and 2208	*ppsA*	P1 10mer-AGAGAAGGGGACCGTCCTG
		P2 10mer-ACCTTGTCCATTTCCGACAC
2337	*ppsA*	P1 10mer-TGGTCTCCGACATGACCGA
		P2 10mer-TTCGCGAGCGATGATCGCC
2551	*trpE*	P1 10mer-GGATCAACGAAGAGGCCGA
		P2 10mer-TCGATCAGCATCAGGTGCTC

P1 = forward amplification primer;

P2 = reverse amplification primer;

10mer = 5′ 10-mer tagACGTTGGATG.

**Table 2:** Extension primers used for the iPLEX20SNP

**SNP site**^**a**^	**Gene target**	**UEP (5′-3′)**	**Mass**	**EP1, mass**	**EP2, mass**	**EP3, mass**	**EP4, mass**
7	*acsA*	ACATCGTCTATGGCCCG	5146.4	C,5393.5	T,5473.5		
45	*acsA*	ggggTCTGTTCGAGGGCGT	5931.8	A,6203	G,6219		
322	*acsA*	gggtaGCGTGGGCGCCCGGCA	6529.2	T,6800.4	C,6816.4		
381	*ascA*	ttcccAGCCGTTCTTCGGCGTGGT	7302.7	C,7549.9	A,7573.9	G,7589.9	T,7629.8
387	*ascA*	gaaGAGGTTGTCCACCAG	5548.6	G,5795.8	A,5875.7		
416^b^	*aroE*	TGCCGTTCAAGGAAGA	4930.2	C,5177.4	A,5201.4	G,5217.4	
		aGgCGTTCAAGGAACT					
488	*aroE*	ccCACCCTGATCCGCCT	5027.3	C,5274.5	G,5314.5	T,5354.4	
881	*aroE*	gagggTGTATGCCAAGGAACCGAC	7451.9	C,7699	G,7739.1	T,7778.9	
894	*guaA*	gTCCTCCAAGGTCCTGCT	5426.5	C,5673.7	A,5697.7	G,5713.7	
937	*guaA*	tgtTCcgCGATGGCCTTGTGCA	6733.4	T,7004.6	C,7020.6		
1086	*guaA*	gtCGAGGACAAGTTCCTCGG	6158	C,6405.2	G,6445.2	T,6485.1	
1152	*guaA*	ctcaGCACCTTCATCGAAGT	6036.9	C,6284.1	G,6324.2	T,6364	
1297	*mutL*	GTGGAAAGCCACGTCGAA	5557.6	T,5828.8	C,5844.8		
1465	*mutL*	gggCGGCtcGCACCTGTGGGG	6520.2	C,6767.4	T,6847.3		
1865	*nuoD*	agagaCAGTCtcGGCACAGTTTCAT	7666	C,7913.2	T,7993.1		
1958	*nuoD*	tgctGATCACGTCGACCCGCTG	6687.3	G,6934.5	C,6974.5		
2169	*ppsA*	ccggaGCGCTGGCCGATGGCACG	7091.6	G,7338.8	T,7362.8	C,7378.8	A,7418.7
2208	*ppsA*	CCGACACGTCGTTGATCAC	5748.7	G,5995.9	A,6075.8		
2337	*ppsA*	CGCCGCGTGGCAGGT	4610	T,4881.2	C,4897.2		
2551	*trpE*	CAGATCCTGCTCCAG	4512.9	G,4760.1	C,4800.2	A,4840	

Non-template bases are indicated in lower case. Mass (Daltons) is provided for the unextended extension primer (UEP), as well as associated extension products (EP) 1, 2 and where relevant 3 and 4.

^a^SNP position is based on the 2882 bp concatenated sequence.

^b^Note that SNP 416 had two extension primers to accommodate a known proximal SNP variation.

Primers and extension primers for each of the 20 SNPs in the iPLEX20SNP were designed as reported previously [14]. All 20 target SNPs were designed for use in a single multiplex well using Assay Designer 4.0 software (Sequenom, Herston, Queensland, Australia). The 24 amplification primers and 21 extension primers used for SNP detection are listed in Tables 1 and 2. Two extension primers with overlapping mass were used for SNP site 416 to accommodate a known proximal SNP variation (Table 2).

SNP detection by MassARRAY was performed as outlined formerly [14], with the following modifications: (1) following the initial PCR, residual PCR Taq polymerase was removed by protease digestion; 1 μl of protease solution (1.07 AU, Qiagen, Doncaster, Victoria, Australia) was added to each PCR reaction and the mixture incubated at 55°C for 30 min followed by an inactivation at 95°C for 5 min; and (2) the single base extension step was performed using the iPLEX Pro Extension Reaction Kit (Sequenom, Herston, Queensland, Australia) following manufacturer’s instructions. SNPs were coded from 1 to 20 to generate a 20 SNP code. The 20-SNP profiles were then interpreted using the data compiled from *in-silico* analysis of the *P. aeruginosa* MLST database, as described below, to provide predicted sequence types (STs). Characterised isolates representative of each SNP were used as reference controls for each test run.

### *In-silico* analysis of 20-SNP profiles from the MLST database

For final result analyses, 1779 concatenated sequences of *P. aeruginosa* housekeeping genes were again downloaded (13^th^ December 2012) and reanalysed. The 1779 *P. aeruginosa* sequences yielded 1401 different STs. The predicted ability of the 20-SNP profile to distinguish these 1401 STs was investigated, as was its ability to distinguish STs of national: AUST-01 (ST- 649), AUST-02 (ST-775), AUST-03 (ST-242), AUST-04 (ST-788), AUST-05 (STs 274 and 781), AUST-06 (ST-801), AUST-07 (ST-262), AUST-08 (STs 782, 783, 784 and 785) and AUST-09 (STs 274 and 1043), AUST-10 (STs 155 and 179), AUST-11 (STs 803, 1034, 1037, 508, 804, 822 and 882), AUST-12 (ST-179), AUST-13 (STs 389 and 800), AUST-14 (STs 155 and 179), AUST-15 (ST-17), AUST-16 (ST-905), AUST-17 (ST-810), AUST-18 (ST-274), AUST-19 (STs 155 and 786), AUST-20 (ST-655), AUST-21 (STs 808), AUST-22 (ST-809), AUST-23 (ST-833), AUST-24 (ST-308), AUST-25 (ST-274), AUST-26 (ST-179), AUST-27 (ST-455), AUST-28 (ST-241), AUST-29 (ST-261), AUST-30 (ST-1036), AUST-31 (ST-274), AUST-32 (ST-236), AUST-33 (ST-12), AUST-34 (ST-1038), AUST-35 (ST-553), AUST-36 (ST-277), AUST-37 (ST-155), AUST-38 (STs 254 and 1041) [15], and international importance: LES (ST-146), Manchester (ST-217), DK2 (ST-386), PA01 (ST-549), PA14 (ST-253), M18 (ST-1239), PACS2 (ST-1394), NCGM2.S1 (ST-235), PA7 (ST-1195), Clone C (ST-17), Dutch-1 (ST-406), Dutch-2 (ST-497) and Midlands (ST-148) [17,19,20].

### Statistical analysis

Discriminatory power and the quantitative measure of congruence between the HRM10SNP and iPLEX20SNP methods and corresponding 95% confidence intervals (CI) were determined by calculating the Simpson’s Index of Diversity and the adjusted Wallace coefficients respectively using the online analysis tool at http://darwin.phyloviz.net/ComparingPartitions/index.php?link=Tool. The 20-SNP profile *in-silico* data were used to predict STs for the 506 clinical isolates utilising the experimental results from the iPLEX20SNP assay.

## Results

### *In-silico* analysis of MLST data

Analysis of the *P. aeruginosa* PubMLST website [17] (13^th^ December 2012) showed that the 1401 STs could be divided into 927 different 20-SNP profiles (Additional file 2: Table S2). Overall, 711 STs could be distinguished individually by the 20-SNP profile, whereas the remaining 690 STs had overlapping 20-SNP profiles with one (n = 120), two (n = 51), three (n = 13), four (n = 11), five (n = 6), six (n = 2), seven (n = 2), eight (n = 1), nine (n = 2), ten (n = 4), 11 (n = 2), 12 (n = 1), or 13 other (n = 1) STs (Additional file 2: Table S2). In total, 486/690 (70.4%) STs showing overlapping 20-SNP profiles comprised closely related single- or double-locus variant STs. Based on these data, the *D*-value for the 20-SNP profiling method was calculated as 0.999 (95% CI 0.998, 0.999). For the STs of national and international importance, nine could be distinguished individually by the 20-SNP profile, whereas the remaining exhibited overlapping 20-SNP profiles. Most of the latter were again single- or double-locus variant STs (Table 3 and Additional file 2: Table S2).

**Table 3:** ***P. aeruginosa *** **MLST data from the *P. aeruginosa *** **MLST database website (13**^**th **^**December 2012) and associated SNP profiles for STs of national and international importance**

**20 SNP profile**^**a**^	**MLST**^**b**^	**Total STs**	**Related STs**
			**(SLV or DLV**^**c**^**)**
CGTCGACTACCTCCCCGGTA	**649(AUST-01)**^**d**^	1	
TGCAAGCTACCTTCCCGGTA	**775(AUST-02)**	1	
TGCAAGCTCCCCTTTGGGCG	**242(AUST-03)**, 996	2	2/2 = SLVs
TGCCGGCTATCCCCCCGACA	787, **788(AUST-04)**	2	2/2 = SLVs
TGTCGGCTACCTTTCGGGTA	209, 268, **274(AUST-05,-09, -18,-25, &-31)** , 466, 546, **781(AUST-05)**, 936, **1043(AUST-09)**, 1068, 1089, 1301, 1326	12	12/12 = SLVs
CGCAAGCTATCCCCCGGGTG	4, **801 (AUST-06)**, 1292	3	2/3 = SLVs
CGCAAGCTATCCCCCCGGCA	**262(AUST-07)**, 774, 1165	3	3/3 = SLVs
CGCAGGGCCCCCTTCGGGCG	**782(AUST-08), 783(AUST-08), 784(AUST-08), 785(AUST-08)**	4	4/4 = SLVs
CGCCGGCTCCCCCCCCGACA	**179(AUST-10,-12,-14,&-26)**, 180, 353	3	3/3 = SLVs
TGCAAGCTACCCCCTGGACA	13, **155(AUST-10,-14, -19, &-37)**, 280, 541, 579, 677, **786(AUST-19)**, 1276, 1316, 1335	10	10/10 = SLVs
CGCAAGCTACCTCCCCGGTA	384, **1037(AUST-11)**	2	
CGCAGGCTACCTCCTGGGTG	**508(AUST-11)**, 937	2	2/2 = SLVs
TGCCGGCTCCCCCCTGGGCA	554, **804(AUST-11)**	2	2/2 = SLVs
CGCAGGCTACCTCCCCGGTA	589, 791, **803(AUST-11)**	3	3/3 = SLVs
TGCCGGCTATCCCCCCGGCA	**822(AUST-11)**, **1239(M18)**	2	2/2 = SLVs
CGCAAGCTACCTCCCCAGTA	**882(AUST-11)**, 1151, 1233	3	3/3 = SLVs
CGCAGGCTACCTTCCCGGTA	**1034(AUST-11)**	1	
CGCAAGGTACCTCCTGGGCG	**800(AUST-13)**	1	
CGCAAGGTCCCCCCCGGGTG	**389(AUST-13)**	1	
CGTCGGCTATCCTTCCGGTA	**17(AUST-15 & Clone C)**, 318, 322, 380, 636, 688, 845, 958, 1255, 1313	10	9/10 = SLVs
CGCAAGCTACTCTCCCGGTG	**905(AUST-16)**, 1039	2	
CGTCGGCTATCCCCTGGGCA	398, 399, 401, **810(AUST-17)**	4	3/4 = SLVs
CGTCGGCCACTCTTCCGGCG	**655(AUST-20)**, 709	2	2/2 = SLVs
TGCAAGCTACCCTCCCGGTA	669, **808(AUST-21)**	2	2/2 = SLVs
CGCCGGGCCTCCTCTGAGTG	**809(AUST-22)**	1	
TGTCGGCTACCTTCCCGGTA	**833(AUST-23)**, 839	2	2/2 = SLVs
TACCAGGCCCCCTCCGAGTG	89, 307, **308(AUST-24)**, 662, 1028	5	2/5 = DLVs
TGTCGGCCCCCCTTCGGGTA	**455(AUST-27)**	1	
CGCAAGCTACCTTCCCGGTA	232, **241(AUST-28),** 247, 379, 471, 577	6	5/6 = SLVs
CGTCGGCTATCCCCTGGGTA	169, **261(AUST-29)**	2	
CGCAGACTCCCCTCCCGGTA	**1036(AUST-30)**	1	
TGCAAGCTATCCTCCCAGCG	**236(AUST-32)**, 239, 240	3	3/3 = SLVs
TGCAAGCTATCCCCCCGGTG	**12(AUST-33)**	1	
CGCAAGCTCCCCCCCGGGTA	103, 244, 441, 462, 464, 594, 766, 986, **1038(AUST-34)**, 1181, 1227, 1338	12	10/12 = SLVs or DLVs
CGCAAGCTATCCTTCCGGTA	445, **553(AUST-35)**	2	
CGCAAGCTCCTCTTTCGGTA	**277(AUST-36)**, 364, 1128, 1390	4	4/4 = SLVs
TGTCGGCTCCTCTTTGGGTA	**254(AUST-38**), **1041(AUST-38)**	2	2/2 = SLVs
TGTCGGCTACCTCCCCGGTG	**146(LES)**, 374, 467, 681, 683, 970	6	6/6 = SLVs
TGCAAGCTACCTCCCCGACG	**217(Manchester)**, 1134	2	2/2 = SLVs
CGCAAGCTACCTCCCCGGCG	**386(DK2)**, 1244	2	
TGCAGGCTCCCCCCCCAGCA	**549(PA01)**, 1331	2	2/2 = SLVs
TATCGGGCCCCCTCCGAGTG	65, 107, 109, **253(PA14)**, 297, 338, 342, 377, 532, 773, 815, 923, 1110, 1363	14	3/14 = SLVs or DLVs
CGTCGGCTCTCCCCTGGACA	**1394(PACS2)**	1	
CGCTAGGCCCCCTCCGAGTG	227, 230, **235(NCGM2.S1)**, 533, 534, 696, 745, 976, 989	9	9/9 = SLVs
CGCGGAGCCCTCTCCGTGTG	366, 368, 1006, 1063, 1190, 1191, **1195(PA7)**	7	4/7 = SLVs
CGTCGGCTACCTCCCCGACA	**406(Dutch-1)**, 484, 519, 536, 547, 575, 608, 1214, 1235, 1312, 1318	11	10/11 = SLVs or DLVs
CGTCGGCTATCCTTTGGGTA	**497(Dutch-2)**, 544, 895, 1317	4	4/4 = SLVs or DLVs
CGCAAGCTATCCCCCGAGTA	138, 140, **148(Midlands)**, 956	4	2/4 = SLVs

The STs consistent with recognised *P. aeruginosa* strains are indicated in parentheses.

^a^SNP profile is in the order of 7, 45, 322, 381, 387, 416, 488, 881, 894, 937, 1086, 1152, 1297, 1465, 1865, 1958, 2169, 2208, 2337 and 2551. These SNPs were derived from the sequence data from the *Pseudomonas aeruginosa* MLST database website (http://darwin.phyloviz.net/ComparingPartitions/index.php?link=Tool) on 13 December 2012. ^b^MLST types available from the *Pseudomonas aeruginosa* MLST database website (http://pubmlst.org/paeruginosa) on 13 December 2012. ^c^SLV: single locus variant, DLV: double locus variant. ^d^Boldface type represents previously characterised National and International MLST types of importance (e.g., AUST-01 has the MLST type 649).

### HRM10SNP and iPLEX20SNP typing of the 506 clinical isolates

Application of the HRM10SNP assay provided complete 10-SNP profiles for 494/506 isolates (type-ability = 97.6%) of which 92 different 10-SNP profiles were observed; 12 isolates were not typed using the HRM10SNP method as one or more SNPs failed to be called by the HRM analysis (Additional file 3: Table S3). The iPLEX20SNP assay provided complete 20-SNP profiles for 471/506 isolates (type-ability = 93.1%) of which there were 147 distinct 20-SNP profiles; 35 isolates failed to provide complete 20-SNP profiles due to the iPLEX20SNP assay failing to characterise one or more SNPs (Additional file 3: Table S3). When the 147 complete 20-SNP profiles (471 isolates) from the iPLEX20SNP assay were used to predict a MLST type (based on the data provided in Additional file 2: Table S2), 124 of 147 (84.4%) profiles matched profiles obtained from the MLST website and there could provide a predicted MLST type or types. Twenty-three 20-SNP profiles from 28 isolates did not match with any of the listed 20-SNP profiles in Additional file 2: Table S2, and therefore a MLST type could not be predicted.

Overall, 470 isolates provided complete SNP profiles by both the HRM10SNP and iPLEX20SNP assays. Simpson’s Index of Diversity and adjusted Wallace coefficients between the HRM10SNP and iPLEX20SNP methods were calculated using these 470 isolates (Table 4). Simpson’s Index of Diversity of the iPLEX20SNP (0.947) was similar to that of the HRM10SNP method (0.944). However, when concordance between the assays was assessed using the adjusted Wallace coefficient, the iPLEX20SNP method (94.9%) was a better predictor of the HRM10SNP method than vice versa (89%). To investigate the latter further we identified all 10-SNP profiles that were further discriminated by the 20-SNP profiles (Additional file 4: Table S4); 34 HRM10SNP profiles were further distinguished into 101 20-SNP profiles using the iPLEX20SNP method. Of note, these involved 30 STs associated with CF strains of local or international importance (Additional file 4: Table S4). In contrast, there were only 11 iPLEX20SNP profiles that were further discriminated by the HRM10SNPassay (Additional file 3: Table S3).

**Table 4:** Number of types, Simpson’s index of diversity and adjusted Wallace coefficients for the HRM10SNP and iPLEX20SNP assays calculated from application to the 470 isolates providing complete SNP profiles by both methods

**Assay**	**No. of types**	**Simpson’s index of diversity**	**Adjusted Wallace coefficient**
		**(95% CI)**	**(95% CI)**
HRM10SNP	91	0.944	0.897
		(0.933-0.955)	(0.876-0.918)
20SNP iPLEX	147	0.947	0.949
		(0.936-0.959)	(0.912-0.986)

Given the high prevalence of AUST-01, AUST-02 and AUST-06 in Australia, and that the HRM10SNP assay was primarily designed to target these strains, we compared the ability of both assays to distinguish these strains. For isolates identified as AUST-01, AUST-02 or AUST-06 by either method (Additional file 3: Table S3), the results of the two methods were in agreement for 80/81 (98.8%), 48/49 (97.8%) and 11/12 (91.7%) isolates, respectively. Both isolates giving discrepant results for AUST-01 and AUST-06 were identified as AUST-01 or AUST-06 by the iPLEX20SNP method, but not by the HRM10SNP assay. For both of these isolates, their 10-SNP profiles by the HRM10SNP differed by only one SNP from the expected profiles of AUST-01 and AUST-06. Upon repeat testing in the HRM10SNP assay, both subsequently typed as AUST-01 and AUST-06, suggesting that there was a mistake in the original HRM10SNP testing. The discordant result for AUST-02 was associated with a different ST; one isolate was identified as AUST-02 by the HRM10SNP method, but differed by two SNPs from the expected 20-SNP profile for AUST-02 in the iPLEX20SNP assay (predicted MLST type of 778).

## Discussion

The *in-silico* analyses of sequence data from the *P. aeruginosa* PubMLST website showed that more than half of recognised STs could be distinguished individually by the 20-SNP profile of the iPLEX20SNP assay. Furthermore, the recognised STs that were unable to be distinguished by this assay were typically single- or double-locus variants. Hence, theoretically the iPLEX20SNP method has considerable potential for broader-based MLST-focused studies of *P. aeruginosa*, here and elsewhere. As the iPLEX20SNP is also based on the Sequenom MassARRAY platform, it is particularly suitable for high-throughput investigations [14]. Using this technology up to 384 isolates can be tested within one working day for less than $AUD 10 per isolate [14], and is therefore quite favourable compared to other technologies. For example, for our 506 test isolates we estimate that classical DNA sequencing-based MLST would have cost approximately $AUS 60,720 ($AUS 120 per isolate), whereas costs for the HRM10SNP and iPLEX20SNP methods were approximately $AUS 10,120 ($AUS 20 per isolate)^13^ and $AUS 5,060 respectively.

Compared to the HRM10SNP, the iPLEX20SNP method clearly provided better discrimination when applied to the *P. aeruginosa* test isolates used in this study. Of note was that the HRM10SNP assay grouped numerous unrelated isolates, including STs of shared strains in the CF patient population, while the iPLEX20SNP method was able to distinguish between these isolates (Additional file 4: Table S4). This was likely due to the higher number of SNPs and that SNP selection for iPLEX20SNP was based on a large international MLST database. These observations provide experimental data to support the above *in-silico* analyses. Indeed, in the clinical context, attaining optimal discriminatory power is particularly important when trying to identify new or emerging shared *P. aeruginosa* strains in CF patients. Consequently, iPLEX20SNP is ideally suited for broader, investigatory studies of *P. aeruginosa* infected patients.

While the HRM10SNP lacked overall discriminatory power, it nevertheless proved to be well-suited for detecting AUST-01, AUST-02 and AUST-06 amongst *P. aeruginosa* isolates from a broad range of Australian CF clinics. AUST-01 and AUST-02 are the shared *P. aeruginosa* strains of greatest concern in Australia [15], and therefore simple methods for detecting these strains remain of local clinical and research interest. The one key benefit of the HRM10SNP method is that it is based on real-time PCR technology, which is now commonplace in most clinical microbiology laboratories. Hence, the HRM10SNP method may still be a useful diagnostic tool locally for laboratories with no access to specialised equipment such as the Sequenom MassARRAY platform.

Limitations in terms of typeability (i.e., the number of isolates providing complete SNP profiles) were observed, however, with 2.4% and 6.9% of isolates failing to give complete profiles in the HRM10SNP and the iPLEX20SNP assays respectively. Typically these problems are caused by poor isolate preparation (i.e., insufficient DNA) or otherwise sequence variation in primer targets [10,14]. Given the sheer diversity amongst the *P. aeruginosa* MLST housekeeping genes, it is highly likely that sequence variation would account for a large proportion of the problems observed here. In any event, we do not see this as an important limitation affecting the broader utility of the assays given that other methods, such as DNA sequencing, could be applied if necessary to the small numbers of untypeable isolates.

## Conclusions

In summary, molecular typing is an integral part of investigating the development and spread of shared *P. aeruginosa* strain genotypes in patients with CF. The iPLEX20SNP is a superior new method providing sufficient throughput and discriminatory power for broader SNP-based MLST-style investigations of *P. aeruginosa*, whereas the HRM10SNP method remains a convenient technique for screening CF clinical isolates for the current most commonly shared Australian *P. aeruginosa* strains and should be able to be performed by most clinical microbiology laboratories.

### Availability of supporting data

The data sets supporting the results of this article are included within the article and its additional files.

## Abbreviations

CI: Confidence interval; CF: Cystic fibrosis; HRM: High-resolution melting; MLST: Multi-locus sequence typing; PCR: Polymerase chain reaction; STs: Sequence types; SNPs: Single nucleotide polymorphisms.

## Competing interests

The authors have no competing interests to declare.

## Authors’ contributions

Conceptualisation by MS, TK, SB, CW, KG, MN, TS, and DW; development by MS, TK, SA, and DW; analysis and interpretation by MS, TK, KR, SA, KG, KMG, DW; the initial drafts were written by MS, TK, KG and DW. All authors provided feedback and have read and approved the final version of the manuscript.

## Authors’ information

Melanie W Syrmis and Timothy J Kidd equal first authors.

## Pre-publication history

The pre-publication history for this paper can be accessed here:

http://www.biomedcentral.com/1471-2334/14/307/prepub

## Supplementary Material

Additional file 1: Table S1 Source and distribution of *Pseudomonas aeruginosa* isolates from cystic fibrosis patients. Click here for file

Additional file 2: Table S2 *P. aeruginosa* MLST data from the *P. aeruginosa* MLST database website (13^th^ December 2012) and associated SNP profiles. iPLEX20SNP predicted STs consistent with recognised *P. aeruginosa* strains are indicated in parentheses. Click here for file

Additional file 3: Table S3 iPLEX20SNP and HRM10SNP results for the 506 isolates. iPLEX20SNP predicted STs or HRM10SNP profiles consistent with recognised *P. aeruginosa* strains are indicated in parentheses. Click here for file

Additional file 4: Table S4 HRM10SNP profiles further discriminated by the iPLEX20SNP assay. iPLEX20SNP predicted STs or HRM10SNP profiles consistent with recognised *P. aeruginosa* strains are indicated in parentheses. Click here for file
