# Supplementary material for: A comparison of two informative SNP-based strategies for typing Pseudomonas aeruginosa isolates from patients with cystic fibrosis
Source: BMC Infect Dis. 2014 Jun 5;14:307. doi: 10.1186/1471-2334-14-307 (PMC4053291; doi:10.1186/1471-2334-14-307)
Supplement: Additional file 1: Table S1 — Source and distribution of Pseudomonas aeruginosa isolates from cystic fibrosis patients. [file 1471-2334-14-307-S1.docx]

**Table S1:** Source and distribution of *Pseudomonas aeruginosa* isolates from cystic

fibrosis patients

| City, state and hospital | Number of isolates |
| --- | --- |
| Adelaide, South Australia |  |
| Royal Adelaide Hospital | 70 |
| Women’s and Children’s Hospital | 24 |
|  |  |
| Brisbane, Queensland |  |
| The Prince Charles Hospital | 71 |
| Royal Children’s Hospital | 30 |
|  |  |
| Melbourne, Victoria |  |
| The Alfred Hospital | 72 |
| Royal Children’s Hospital | 31 |
| Monash Medical Centre | 16 |
|  |  |
| Perth, Western Australia |  |
| Sir Charles Gairdner Hospital | 70 |
| Princess Margaret Hospital for Children | 20 |
|  |  |
| Sydney, New South Wales |  |
| Royal Prince Alfred Hospital | 72 |
| Children’s Hospital Westmead | 30 |
