# Supplementary material for: A comparison of two informative SNP-based strategies for typing Pseudomonas aeruginosa isolates from patients with cystic fibrosis
Source: BMC Infect Dis. 2014 Jun 5;14:307. doi: 10.1186/1471-2334-14-307 (PMC4053291; doi:10.1186/1471-2334-14-307)
Supplement: Additional file 2: Table S2 — P. aeruginosa MLST data from the P. aeruginosa MLST database website (13th December 2012) and associated SNP profiles. iPLEX20SNP predicted STs consistent with recognised P. aeruginosa strains are indicated in parentheses. [file 1471-2334-14-307-S2.docx]

**Table S2.** *P. aeruginosa* MLST data from the *P. aeruginosa* MLST database website (13^th^ December 2012) and associated SNP profiles. iPLEX20SNP predicted STs consistent with recognised *P. aeruginosa* strains are indicated in parentheses.

| 20 SNP profile^a^ | MLST ^b^ | Total STs | Related STs  (SLV or DLV^c^) |
| --- | --- | --- | --- |
| AGCCGGCTCCCTTCCCGGTA | 220 | 1 |  |
| CATCGGCCCCTCCCCGAGCG | 1200 | 1 |  |
| CATCGGCCCCTCCCCGAGTG | 1021 | 1 |  |
| CATCGGCCCCTCTCCGAGTG | 478 | 1 |  |
| CATCGGCTCCTCCCCGAGTA | 813 | 1 |  |
| CATCGGCTCCTCTCCGAGTG | 1359 | 1 |  |
| CATCGGGCCCCCCCCGAGTG | 365, 922 | 2 | 2/2 = SLVs |
| CATCGGGCCCTCCCCGAGTG | 1114 | 1 |  |
| CATCGGGCCCTCTCCGAGCG | 945 | 1 |  |
| CATCGGGCCCTCTCCGAGTG | 10 | 1 |  |
| CATCGGGTCCCCCCCGAGTG | 522 | 1 |  |
| CGCAAACCCCCCTCCGAGTG | 1369 | 1 |  |
| CGCAAACTCCCCTCTGGGCG | 143 | 1 |  |
| CGCAAACTCTCCTTCCAGCA | 5 | 1 |  |
| CGCAAGCCACCCTTTGGGCA | 142 | 1 |  |
| CGCAAGCCACCTTTCGAGTG | 476 | 1 |  |
| CGCAAGCCACCTTTCGGGCA | 388 | 1 |  |
| CGCAAGCCACTCCCTGAGTA | 35 | 1 |  |
| CGCAAGCCACTCTCCGAGTG | 479 | 1 |  |
| CGCAAGCCCCCCCCCCAGTA | 908, 940 | 2 |  |
| CGCAAGCCCCCCCCTGGGTA | 1378 | 1 |  |
| CGCAAGCCCCCCCTTGGGTA | 469 | 1 |  |
| CGCAAGCCCCCCTTCGGGCA | 426 | 1 |  |
| CGCAAGCCCCCCTTCGGGCG | 1111 | 1 |  |
| CGCAAGCCCCCCTTCGGGTA | 1311 | 1 |  |
| CGCAAGCCCCCCTTTGGGCA | 245 | 1 |  |
| CGCAAGCCCCTCTTCCAGTA | 90, 1374 | 2 | 2/2 = SLVs |
| CGCAAGCCCCTCTTTGGGCA | 740 | 1 |  |
| CGCAAGCTACCCCCCCGGTA | 884, 1129 | 2 |  |
| CGCAAGCTACCCCCCGGGTA | 1161 | 1 |  |
| CGCAAGCTACCCCCCGGGTG | 948 | 1 |  |
| CGCAAGCTACCCCCTCAGTA | 1398 | 1 |  |
| CGCAAGCTACCCCCTGAGTG | 299 | 1 |  |
| CGCAAGCTACCCCCTGGACA | 210 | 1 |  |
| CGCAAGCTACCCCCTGGGTG | 904 | 1 |  |
| CGCAAGCTACCCTCCCGGCA | 903 | 1 |  |
| CGCAAGCTACCCTCCCGGTG | 1062 | 1 |  |
| CGCAAGCTACCCTCCGGGCA | 1393 | 1 |  |
| CGCAAGCTACCCTCCGGGTA | 670 | 1 |  |
| CGCAAGCTACCCTTCCAGCA | 144 | 1 |  |
| CGCAAGCTACCCTTCGGGTA | 892 | 1 |  |
| CGCAAGCTACCTCCCCAGTA | **882(AUST-11)**, 1151, 1233 | 3 | 3/3 = SLVs |
| CGCAAGCTACCTCCCCGACA | 1003 | 1 |  |
| CGCAAGCTACCTCCCCGGCG | **386(DK2)**, 1244 | 2 |  |
| CGCAAGCTACCTCCCCGGTA | 384, **1037(AUST-11)** | 2 |  |
| CGCAAGCTACCTCCCCGGTG | 394 | 1 |  |
| CGCAAGCTACCTCCCGAGTA | 371, 703 | 2 |  |
| CGCAAGCTACCTCCCGGGTA | 592, 998 | 2 |  |
| CGCAAGCTACCTCCCGGGTG | 106, 893 | 2 |  |
| CGCAAGCTACCTCCTCAGTG | 812 | 1 |  |
| CGCAAGCTACCTCCTGAGTG | 790, 981 | 2 | 2/2 = DLVs |
| CGCAAGCTACCTCCTGGGTA | 272, 348, 416, 1170, 1213, 1320 | 6 | 6/6 = SLVs |
| CGCAAGCTACCTCCTGGGTG | 789 | 1 |  |
| CGCAAGCTACCTTCCCAGTA | 886 | 1 |  |
| CGCAAGCTACCTTCCCGGCA | 689 | 1 |  |
| CGCAAGCTACCTTCCCGGCG | 685 | 1 |  |
| CGCAAGCTACCTTCCCGGTA | 232, **241(AUST-28),** 247, 379, 471, 577 | 6 | 5/6 = SLVs |
| CGCAAGCTACCTTCCGGGCG | 93 | 1 |  |
| CGCAAGCTACCTTCTGAGTA | 1055, 1058 | 2 | 2/2 = SLVs |
| CGCAAGCTACCTTCTGGGTA | 734, 883 | 2 | 2/2 = DLVs |
| CGCAAGCTACCTTTCCGGTG | 403 | 1 |  |
| CGCAAGCTACCTTTCGAGTG | 939 | 1 |  |
| CGCAAGCTACCTTTCGGGCA | 1234 | 1 |  |
| CGCAAGCTACCTTTTGGGCA | 1057 | 1 |  |
| CGCAAGCTACTCCCCGAGTA | 568 | 1 |  |
| CGCAAGCTACTCCCCGGGTA | 630 | 1 |  |
| CGCAAGCTACTCTCCCGGTG | **905(AUST-16)**, 1039 | 2 |  |
| CGCAAGCTACTCTTCCGGCG | 902 | 1 |  |
| CGCAAGCTATCCCCCCAGTA | 1144 | 1 |  |
| CGCAAGCTATCCCCCCGGCA | **262(AUST-07)**, 774, 1165 | 3 | 3/3 = SLVs |
| CGCAAGCTATCCCCCCGGTG | 229,390, 928 | 3 | 3/3 = SLVs |
| CGCAAGCTATCCCCCGAGTA | 138, 140, **148(Midlands)**, 956 | 4 | 2/4 = SLVs |
| CGCAAGCTATCCCCCGGGTG | 4, **801 (AUST-06)**, 1292 | 3 | 2/3 = SLVs |
| CGCAAGCTATCCCCTGAGCG | 504 | 1 |  |
| CGCAAGCTATCCCCTGGACG | 962 | 1 |  |
| CGCAAGCTATCCCCTGGGTA | 891, 1243 | 2 |  |
| CGCAAGCTATCCTCCCGGTG | 819 | 1 |  |
| CGCAAGCTATCCTCCGGGTG | 1095, 1193 | 2 | 2/2 = SLVs |
| CGCAAGCTATCCTTCCGGTA | 445, **553(AUST-35)** | 2 |  |
| CGCAAGCTATCCTTCGAGTA | 952 | 1 |  |
| CGCAAGCTATCCTTTGGGTA | 602 | 1 |  |
| CGCAAGCTCCCCCCCCAGTG | 1065 | 1 |  |
| CGCAAGCTCCCCCCCCGGTA | 597 | 1 |  |
| CGCAAGCTCCCCCCCCGGTG | 111, 113, 284, 584, 600, 772, 1314, 1319 | 8 | 8/8 = SLVs or DLVs |
| CGCAAGCTCCCCCCCGAATG | 890 | 1 |  |
| CGCAAGCTCCCCCCCGGGTA | 103, 244, 441, 462, 464, 594, 766, 986, **1038(AUST-34)**, 1181, 1227, 1338 | 12 | 10/12 = SLVs or DLVs |
| CGCAAGCTCCCCCCCGGGTG | 30, 121 | 2 |  |
| CGCAAGCTCCCCCCTGGGTA | 98, 1119 | 2 | 2/2 = DLVs |
| CGCAAGCTCCCCTCCCAGTA | 960 | 1 |  |
| CGCAAGCTCCCCTCCCGGCG | 885 | 1 |  |
| CGCAAGCTCCCCTCCCGGTG | 302 | 1 |  |
| CGCAAGCTCCCCTCCGAGCG | 91 | 1 |  |
| CGCAAGCTCCCCTCCGGGCG | 47, 1375 | 2 |  |
| CGCAAGCTCCCCTCCGGGTA | 123, 595, 1344 | 3 | 2/3 = SLVs |
| CGCAAGCTCCCCTCTCGGTA | 1272 | 1 |  |
| CGCAAGCTCCCCTTCCAGTG | 767 | 1 |  |
| CGCAAGCTCCCCTTCCGGTA | 901 | 1 |  |
| CGCAAGCTCCCCTTCCGGTG | 611, 1075, 1273 | 3 |  |
| CGCAAGCTCCCCTTTGGGCA | 561, 580, 1217 | 3 | 3/3 = SLVs |
| CGCAAGCTCCCCTTTGGGCG | 888 | 1 |  |
| CGCAAGCTCCCTCCTGAGTA | 172 | 1 |  |
| CGCAAGCTCCCTTTTCAGTG | 1126 | 1 |  |
| CGCAAGCTCCTCCCCCAGTG | 741 | 1 |  |
| CGCAAGCTCCTCCCCCGGTA | 1061 | 1 |  |
| CGCAAGCTCCTCCCCCGGTG | 654, 964 | 2 | 2/2 = SLVs |
| CGCAAGCTCCTCCCCGAGTG | 605, 1186 | 2 |  |
| CGCAAGCTCCTCCCCGGGCA | 358 | 1 |  |
| CGCAAGCTCCTCCCCGGGTG | 1309 | 1 |  |
| CGCAAGCTCCTCCCTCGGTA | 659 | 1 |  |
| CGCAAGCTCCTCTCCCGGTA | 1046 | 1 |  |
| CGCAAGCTCCTCTCCGAGTG | 75, 108, 425, 581 | 4 | 3/4 = SLVs |
| CGCAAGCTCCTCTCTCGGTA | 758 | 1 |  |
| CGCAAGCTCCTCTTTCGGTA | **277(AUST-36)**, 364, 1128, 1390 | 4 | 4/4 = SLVs |
| CGCAAGCTCCTCTTTCGGTG | 1252 | 1 |  |
| CGCAAGCTCTCCCCCCGGCG | 647, 1168 | 2 | 2/2 = SLVs |
| CGCAAGCTCTCCCCCCGGTA | 889 | 1 |  |
| CGCAAGCTCTCCCCCCGGTG | 283, 887 | 2 |  |
| CGCAAGCTCTCCTTCCAGTA | 700 | 1 |  |
| CGCAAGCTCTCCTTCCGGTG | 219 | 1 |  |
| CGCAAGCTCTCCTTTCGGTA | 206 | 1 |  |
| CGCAAGCTCTCTCCCGGGTA | 408 | 1 |  |
| CGCAAGCTCTCTTCCGAGTA | 1124 | 1 |  |
| CGCAAGCTCTCTTCCGAGTG | 1059 | 1 |  |
| CGCAAGCTGCCGCCTGGGTA | 1136 | 1 |  |
| CGCAAGGCACCCCCTGGACA | 1388 | 1 |  |
| CGCAAGGCACCTCCCCGGTA | 1159 | 1 |  |
| CGCAAGGCACCTCCTGGGCG | 983 | 1 |  |
| CGCAAGGCACTCTCTGGGCA | 270 | 1 |  |
| CGCAAGGCACTCTTCCGACA | 88 | 1 |  |
| CGCAAGGCATCCTTCCGGTA | 1009, 1067 | 2 | 2/2 = SLVs |
| CGCAAGGCCCCCCCCCAGCA | 881 | 1 |  |
| CGCAAGGCCCCCCCCCGGTG | 966 | 1 |  |
| CGCAAGGCCCCCCCCGAGCA | 931 | 1 |  |
| CGCAAGGCCCCCCCCGAGTG | 623, 1147 | 2 | 2/2 = SLVs |
| CGCAAGGCCCCCTCCGAGTG | 1004, 1242, 1304 | 3 |  |
| CGCAAGGCCCCCTTCGGGCG | 1000 | 1 |  |
| CGCAAGGCCCTCCCCGAGTG | 76, 337 | 2 |  |
| CGCAAGGCCTCCCCCCAGCA | 1223 | 1 |  |
| CGCAAGGTACCCCCCGAGTA | 1290 | 1 |  |
| CGCAAGGTACCTCCCCGGTA | 451 | 1 |  |
| CGCAAGGTACCTCCTGAGTG | 880 | 1 |  |
| CGCAAGGTACCTCCTGGGCG | **800(AUST-13)** | 1 |  |
| CGCAAGGTACCTTTCGGGTG | 599 | 1 |  |
| CGCAAGGTACCTTTTGAGTG | 251 | 1 |  |
| CGCAAGGTACCTTTTGGGTG | 894 | 1 |  |
| CGCAAGGTATCCCCCGGGTG | 424 | 1 |  |
| CGCAAGGTATCCCCTGGGTG | 818 | 1 |  |
| CGCAAGGTCCCCCCCCGGTG | 1380 | 1 |  |
| CGCAAGGTCCCCCCCGGGTG | **389(AUST-13)** | 1 |  |
| CGCAAGGTCCCCCCTGGGTA | 99 | 1 |  |
| CGCAAGGTCCCCTCCGAGTG | 1370 | 1 |  |
| CGCAAGGTCCCCTCCGGGTG | 77 | 1 |  |
| CGCAAGGTCCCCTTTGAGCG | 794 | 1 |  |
| CGCAAGGTCCCTCCTGGGCG | 799 | 1 |  |
| CGCAAGGTCCTCTTCCAGCG | 750 | 1 |  |
| CGCAAGGTCTCCCCCCAGCA | 501 | 1 |  |
| CGCAAGTTCTCTTCCCAGTA | 271 | 1 |  |
| CGCAAGTTCTCTTCTGAGTG | 697 | 1 |  |
| CGCAGACCCCGCCCCGGGTG | 200, 215, 637, 679, 705, 947, 955, 1045, 1198, 1225, 1328 | 11 | 4/11 = SLVs or DLVs |
| CGCAGACCCCGCCCTGGGTG | 1201 | 1 |  |
| CGCAGACCCCGCTTCGGGTG | 1137 | 1 |  |
| CGCAGACCCCTCCCCGGGTG | 222 | 1 |  |
| CGCAGACTACCTCCCGGGTG | 545 | 1 |  |
| CGCAGACTCCCCTCCCGACG | 1241 | 1 |  |
| CGCAGACTCCCCTCCCGGTA | **1036(AUST-30)** | 1 |  |
| CGCAGACTCCCCTCCGGACG | 506 | 1 |  |
| CGCAGACTCCGCCCCGGGTG | 517 | 1 |  |
| CGCAGACTCCTCCCCGGGTG | 1172 | 1 |  |
| CGCAGGCCCCCCCCCCGGTG | 127 | 1 |  |
| CGCAGGCCCCCCCCCGGGTG | 963, 1348 | 2 |  |
| CGCAGGCCCCCCTCCCGGTA | 920 | 1 |  |
| CGCAGGCCCCTCCCCGGGTG | 195, 224, 349, 359, 719, 961, 977, 1077, 1183, 1221, 1222, 1266, 1278 | 13 | 11/13 = SLVs or DLVs |
| CGCAGGCTACCCCCCCAGTG | 1230 | 1 |  |
| CGCAGGCTACCCCCCCGGTA | 1032 | 1 |  |
| CGCAGGCTACCCTCCCGGCG | 1104 | 1 |  |
| CGCAGGCTACCCTCCCGGTA | 1395 | 1 |  |
| CGCAGGCTACCCTTTCGGTA | 360, 861, 862, 864 | 4 | 4/4 = SLVs |
| CGCAGGCTACCCTTTGGGTA | 869 | 1 |  |
| CGCAGGCTACCCTTTGGGTG | 867, 1302 | 2 | 2/2 = SLVs |
| CGCAGGCTACCTCCCCGACG | 950 | 1 |  |
| CGCAGGCTACCTCCCCGGCA | 866 | 1 |  |
| CGCAGGCTACCTCCCCGGTA | 589, 791, **803(AUST-11)** | 3 | 3/3 = SLVs |
| CGCAGGCTACCTCCCCGGTG | 562 | 1 |  |
| CGCAGGCTACCTCCCGAGTG | 20 | 1 |  |
| CGCAGGCTACCTCCTGAGTA | 28 | 1 |  |
| CGCAGGCTACCTCCTGGGTG | **508(AUST-11)**, 937 | 2 | 2/2 = SLVs |
| CGCAGGCTACCTTCCCGGTA | **1034(AUST-11)** | 1 |  |
| CGCAGGCTACCTTCTGAGTG | 427 | 1 |  |
| CGCAGGCTACCTTTCCAGTA | 287, 288 | 2 | 2/2 = SLVs |
| CGCAGGCTACCTTTCCGGTA | 865 | 1 |  |
| CGCAGGCTACCTTTTGGGTA | 863 | 1 |  |
| CGCAGGCTACTCCCCCAGTA | 953 | 1 |  |
| CGCAGGCTACTCTCCCGGCA | 43 | 1 |  |
| CGCAGGCTATCCCCCCGGTG | 1098 | 1 |  |
| CGCAGGCTATCCCCCGGGTA | 918 | 1 |  |
| CGCAGGCTATCCCCTGAGTA | 363 | 1 |  |
| CGCAGGCTATCCCCTGGGTA | 110, 1199 | 2 | 2/2= SLVs |
| CGCAGGCTATCCTCCGGGTG | 1145 | 1 |  |
| CGCAGGCTATCCTTCCGGTG | 919 | 1 |  |
| CGCAGGCTCCCCCCCCGGTG | 102, 814 | 2 | 2/2 = DLVs |
| CGCAGGCTCCCCTCCCGGTA | 385 | 1 |  |
| CGCAGGCTCCCCTCCCGGTG | 443 | 1 |  |
| CGCAGGCTCCCCTTCGGGTA | 898 | 1 |  |
| CGCAGGCTCCCTCCCCGGTG | 1005 | 1 |  |
| CGCAGGCTCCGCCCCGGGTA | 965 | 1 |  |
| CGCAGGCTCCGCCCCGGGTG | 528 | 1 |  |
| CGCAGGCTCCTCCCCGGGTA | 1174 | 1 |  |
| CGCAGGCTCCTCCCCGGGTG | 516, 574, 586, 1173 | 4 | 2/4 = DLVs |
| CGCAGGCTCCTCTCCGGGTG | 621 | 1 |  |
| CGCAGGCTCCTCTCTGAGCA | 938 | 1 |  |
| CGCAGGCTCTCCCCCGAGTA | 868 | 1 |  |
| CGCAGGCTCTCCCCTGAGTA | 1247 | 1 |  |
| CGCAGGGCACCCTCCCGGCG | 760 | 1 |  |
| CGCAGGGCACCTTTCGAGTG | 1231 | 1 |  |
| CGCAGGGCCCCCCCCCGGTG | 1347 | 1 |  |
| CGCAGGGCCCCCTTCGGGCG | **782(AUST-08), 783(AUST-08), 784(AUST-08), 785(AUST-08)** | 4 | 4/4 = SLVs |
| CGCAGGGCCCTCTCCCGGTG | 130 | 1 |  |
| CGCAGGGCCCTCTCCGAGTG | 732 | 1 |  |
| CGCAGGGTACCTCCTGGGTG | 698 | 1 |  |
| CGCAGGGTACCTTTCGAGTG | 535 | 1 |  |
| CGCAGGGTACCTTTCGGGCG | 490 | 1 |  |
| CGCAGGGTATCCTTCCGGTG | 1122 | 1 |  |
| CGCAGGGTCCCCCCTGGGTG | 951 | 1 |  |
| CGCAGGGTCCCCTCTGAGTG | 1080, 1123 | 2 | 2/2 = SLVs |
| CGCCAGCTATCTCCCCGGTG | 396, 397 | 2 | 2/2= SLVs |
| CGCCAGCTCCCCCCCGGGCA | 1357 | 1 |  |
| CGCCAGCTCCCCTCCGAGTG | 94, 1355 | 2 |  |
| CGCCAGGCCCCCCCCCGGTG | 124 | 1 |  |
| CGCCAGGCCCCCCCCGAGTG | 1002 | 1 |  |
| CGCCAGGCCCCCTCCGAGTG | 1352 | 1 |  |
| CGCCGGCCCCCCTTCGGGCG | 378, 638, 797 | 3 | 3/3 = SLVs |
| CGCCGGCTACCCCCCCGACG | 717 | 1 |  |
| CGCCGGCTACCCCCCCGGTG | 827 | 1 |  |
| CGCCGGCTACCCTCTGAGTA | 825 | 1 |  |
| CGCCGGCTACCCTTTCAGTA | 1113 | 1 |  |
| CGCCGGCTACCTCCCCAGTG | 754 | 1 |  |
| CGCCGGCTACCTCCCCGGCA | 707 | 1 |  |
| CGCCGGCTACCTCCCCGGCG | 234 | 1 |  |
| CGCCGGCTACCTCTTCAGTA | 1103 | 1 |  |
| CGCCGGCTACCTTCCCGGCG | 1050 | 1 |  |
| CGCCGGCTACCTTCCCGGTA | 1157 | 1 |  |
| CGCCGGCTACCTTCCGAGTA | 1154 | 1 |  |
| CGCCGGCTACCTTTCCGGCA | 170, 367, 373, 997, 1315 | 5 | 5/5 = SLVs |
| CGCCGGCTACCTTTTCGGCA | 652 | 1 |  |
| CGCCGGCTACCTTTTGGGCA | 1171 | 1 |  |
| CGCCGGCTATCCCCCCAGTA | 494 | 1 |  |
| CGCCGGCTATCCCCCCGGCG | 512 | 1 |  |
| CGCCGGCTATCCCCCCGGTG | 556 | 1 |  |
| CGCCGGCTATCCCCTGAGTG | 796 | 1 |  |
| CGCCGGCTATCCTCCCGGCG | 530 | 1 |  |
| CGCCGGCTATCCTCCCGGTG | 826 | 1 |  |
| CGCCGGCTATCCTTCCGATA | 629 | 1 |  |
| CGCCGGCTATCCTTCCGGCG | 164 | 1 |  |
| CGCCGGCTATCCTTCCGGTG | 1274 | 1 |  |
| CGCCGGCTATCTCCCCGGTG | 133, 482, 513, 514 | 4 | 4/4 = SLVs |
| CGCCGGCTCCCCCCCCGACA | **179(AUST-10,-12,-14,&-26)**, 180, 353 | 3 | 3/3 = SLVs |
| CGCCGGCTCCCCCCCCGACG | 1088 | 1 |  |
| CGCCGGCTCCCCCCCCGGCA | 188 | 1 |  |
| CGCCGGCTCCCCCCCCGGTA | 178, 1305 | 2 | 2/2 = SLVs |
| CGCCGGCTCCCCCCTGGACA | 203 | 1 |  |
| CGCCGGCTCCCCTCCCGACA | 158 | 1 |  |
| CGCCGGCTCCCCTTCCAGTA | 1007 | 1 |  |
| CGCCGGCTCCCTTCCCGGTA | 165, 192, 1389 | 3 | 3/3 = SLVs |
| CGCCGGCTCCCTTCTGGGTA | 1079 | 1 |  |
| CGCCGGCTCCTCCCCGGGCG | 1342 | 1 |  |
| CGCCGGCTCCTCTCTGGGTA | 369 | 1 |  |
| CGCCGGGCACCTTCCCGGCG | 1297 | 1 |  |
| CGCCGGGCATCCTCCGAGCG | 1 | 1 |  |
| CGCCGGGCATCCTCCGGACG | 1106 | 1 |  |
| CGCCGGGCCCCCTCCGAGTA | 197 | 1 |  |
| CGCCGGGCCCCCTCCGAGTG | 125 | 1 |  |
| CGCCGGGCCTCCTCTGAGTG | **809(AUST-22)** | 1 |  |
| CGCCGGGTACCTTTCCGGCA | 949 | 1 |  |
| CGCGCCCTGCCCCCCGCGTC | 725 | 1 |  |
| CGCGCTCTGCCCCCCGCGTC | 727 | 1 |  |
| CGCGGAGCACCTTCCGTGTG | 372 | 1 |  |
| CGCGGAGCCCTCTCCGTGTA | 1120 | 1 |  |
| CGCGGAGCCCTCTCCGTGTG | 366, 368, 1006, 1063, 1190, 1191, **1195(PA7)** | 7 | 4/7 = SLVs |
| CGCGGCCGGCCCCCCGCGTC | 715 | 1 |  |
| CGCGGCCTCCCCCCCGGGTC | 722 | 1 |  |
| CGCGGCCTGCCCCCCGCGTC | 710,711, 723, 724, 726 | 5 |  |
| CGCGGGCTACCTCCCCGGCG | 1385 | 1 |  |
| CGCGGGCTCCTCTCCGTGTA | 911, 912 | 2 |  |
| CGCGGGCTCCTCTCCGTGTG | 191 | 1 |  |
| CGCGGGCTGCCTCCCGCGTA | 714 | 1 |  |
| CGCGGGGTCCCGTTCGAGTG | 610 | 1 |  |
| CGCTAGCTCCCCCCCGGGCA | 51 | 1 |  |
| CGCTAGCTCCCCTCCGAGTA | 1307 | 1 |  |
| CGCTAGCTCCCCTCCGAGTG | 49, 593 | 2 |  |
| CGCTAGCTCCCTCCTGAGTA | 1084 | 1 |  |
| CGCTAGGCACCCCCCGAGTG | 454 | 1 |  |
| CGCTAGGCACCCTCCGAGTG | 265, 457 | 2 |  |
| CGCTAGGCCCCCCCCGAGTG | 1086 | 1 |  |
| CGCTAGGCCCCCCCCGGGCG | 86 | 1 |  |
| CGCTAGGCCCCCCCTGAGTG | 971 | 1 |  |
| CGCTAGGCCCCCTCCCAGTG | 1015, 1162 | 2 | 2/2 = SLVs |
| CGCTAGGCCCCCTCCGAGTG | 227, 230, **235(NCGM2.S1)**, 533, 534, 696, 745, 976, 989 | 9 | 9/9 = SLVs |
| CGCTAGGCCCCCTCCGGACG | 79, 141 | 2 | 2/2 = DLVs |
| CGCTAGGCCCCCTCCGGGTG | 657, 1081 | 2 | 2/2 = SLVs |
| CGCTAGGCCCCCTCTGAGTG | 651 | 1 |  |
| CGCTAGGCCCCCTTCGAGTG | 660 | 1 |  |
| CGCTAGGCCCCTTCCGAGTG | 248 | 1 |  |
| CGCTAGGCCCTCCCCGAGTG | 327 | 1 |  |
| CGCTAGGCCCTCTCCGAGCG | 301 | 1 |  |
| CGCTAGGCCCTCTCCGAGTG | 1140 | 1 |  |
| CGCTAGGCCTCCTCCGAGTG | 824 | 1 |  |
| CGCTAGGTCCCCTCCGAGTG | 622 | 1 |  |
| CGCTAGGTCCCCTTCGAGTG | 1300 | 1 |  |
| CGCTGACCCCCCCCCGGGCG | 913 | 1 |  |
| CGCTGGCTCCCCTCCCGACA | 1387 | 1 |  |
| CGTAAGCTACCTCCCCGGTG | 292, 946 | 2 |  |
| CGTAAGCTATCCCCCCGGTG | 189 | 1 |  |
| CGTAAGCTATCCTCCCAGTA | 225 | 1 |  |
| CGTAAGCTCCCCCCCGGGTG | 1349 | 1 |  |
| CGTAAGGTACCTTCTGGGTA | 1356 | 1 |  |
| CGTCAGCTATCCTTTGGGTA | 576 | 1 |  |
| CGTCGACCACCTCCCGGGTG | 1351 | 1 |  |
| CGTCGACCCTCCCCCCGGTA | 6 | 1 |  |
| CGTCGACTACCTCCCCGGCA | 675 | 1 |  |
| CGTCGACTACCTCCCCGGTA | **649(AUST-01)** | 1 |  |
| CGTCGACTCCTCTTTCGGTA | 1069 | 1 |  |
| CGTCGGCCACCTCCCCGGTG | 435 | 1 |  |
| CGTCGGCCACCTCCTCGGTG | 1226 | 1 |  |
| CGTCGGCCACCTTTTGAGTG | 432 | 1 |  |
| CGTCGGCCACTCCCCCGGTG | 430 | 1 |  |
| CGTCGGCCACTCTTCCGGCG | **655(AUST-20)**, 709 | 2 | 2/2 = SLVs |
| CGTCGGCCCCCCTTTGAGTG | 673 | 1 |  |
| CGTCGGCCCTCCCCTGAGTG | 419 | 1 |  |
| CGTCGGCCCTCCCCTGGGTG | 856 | 1 |  |
| CGTCGGCTACCCCCCCAGTG | 942 | 1 |  |
| CGTCGGCTACCCCCCGAGTA | 226, 598, 847, 896, 1087, 1237 | 6 | 5/6 = SLVs |
| CGTCGGCTACCCCCTCGGCG | 161 | 1 |  |
| CGTCGGCTACCCTCTGGGTA | 166, 1156 | 2 |  |
| CGTCGGCTACCCTTTGGGTA | 183, 1215 | 2 | 2/2 = SLVs |
| CGTCGGCTACCTCCCCAGTG | 1238 | 1 |  |
| CGTCGGCTACCTCCCCGACA | **406(Dutch-1)**, 484, 519, 536, 547, 575, 608, 1214, 1235, 1312, 1318 | 11 | 10/11 = SLVs or DLVs |
| CGTCGGCTACCTCCCCGGCA | 460 | 1 |  |
| CGTCGGCTACCTCCCCGGCG | 259, 1295, 1392 | 3 | 2/3 = SLVs |
| CGTCGGCTACCTCCCCGGTA | 1070, 1177 | 2 | 2/2 = DLVs |
| CGTCGGCTACCTCCCGAGCA | 447 | 1 |  |
| CGTCGGCTACCTCCCGAGCG | 448 | 1 |  |
| CGTCGGCTACCTCCCGAGTG | 480 | 1 |  |
| CGTCGGCTACCTCCCGGGTG | 36, 402 | 2 |  |
| CGTCGGCTACCTCCTGAGTG | 1189 | 1 |  |
| CGTCGGCTACCTCCTGGGCG | 1212 | 1 |  |
| CGTCGGCTACCTTCCCAGTA | 167 | 1 |  |
| CGTCGGCTACCTTCCCGACA | 405, 987 | 2 |  |
| CGTCGGCTACCTTCCCGGCG | 1035 | 1 |  |
| CGTCGGCTACCTTCCCGGTA | 653, 934 | 2 | 2/2 = DLVs |
| CGTCGGCTACCTTCCCGGTG | 739 | 1 |  |
| CGTCGGCTACCTTCCGGGCG | 1218 | 1 |  |
| CGTCGGCTACCTTCTGAGTA | 258, 626 | 2 | 2/2 = SLVs |
| CGTCGGCTACCTTCTGGGCG | 859 | 1 |  |
| CGTCGGCTACCTTCTGGGTG | 1125 | 1 |  |
| CGTCGGCTACCTTTCCAGTA | 916 | 1 |  |
| CGTCGGCTACCTTTCCAGTG | 848 | 1 |  |
| CGTCGGCTACCTTTCCGACA | 523 | 1 |  |
| CGTCGGCTACCTTTCCGACG | 849 | 1 |  |
| CGTCGGCTACCTTTCCGGCG | 957 | 1 |  |
| CGTCGGCTACCTTTCCGGTA | 693 | 1 |  |
| CGTCGGCTACCTTTCGGGTA | 276 | 1 |  |
| CGTCGGCTACCTTTTCAGTA | 1207 | 1 |  |
| CGTCGGCTACCTTTTGAGTA | 198, 1048 | 2 | 2/2 = SLVs |
| CGTCGGCTACCTTTTGAGTG | 529, 569, 857 | 3 | 3/3 = SLVs or DLVs |
| CGTCGGCTACCTTTTGGGCG | 858 | 1 |  |
| CGTCGGCTACTCCCCCGGTA | 639, 853 | 2 | 2/2 = SLVs |
| CGTCGGCTACTCCCCCGGTG | 53 | 1 |  |
| CGTCGGCTACTCCCTGGGTA | 1054 | 1 |  |
| CGTCGGCTACTCTCCCGGCA | 347 | 1 |  |
| CGTCGGCTACTCTTCCAGTA | 820 | 1 |  |
| CGTCGGCTACTCTTCCGGCG | 738 | 1 |  |
| CGTCGGCTATCCCCCCAGTG | 97 | 1 |  |
| CGTCGGCTATCCCCCCGGCA | 400 | 1 |  |
| CGTCGGCTATCCCCCCGGTA | 202 | 1 |  |
| CGTCGGCTATCCCCCCGGTG | 802 | 1 |  |
| CGTCGGCTATCCCCCGAGTA | 83, 492, 682 | 3 | 2/3 = DLVs |
| CGTCGGCTATCCCCCGAGTG | 474 | 1 |  |
| CGTCGGCTATCCCCCGGGTA | 846 | 1 |  |
| CGTCGGCTATCCCCCGGGTG | 92, 218, 1376 | 3 | 2/3 = SLVs |
| CGTCGGCTATCCCCTGGGCA | 398, 399, 401, **810(AUST-17)** | 4 | 3/4 = SLVs |
| CGTCGGCTATCCCCTGGGTA | 169, **261(AUST-29)** | 2 |  |
| CGTCGGCTATCCCCTGGGTG | 392, 590, 733 | 3 | 2/3 = SLVs |
| CGTCGGCTATCCTCCCGGTA | 281, 954 | 2 | 2/2 = SLVs |
| CGTCGGCTATCCTCCGGGTG | 57 | 1 |  |
| CGTCGGCTATCCTTCCGGTA | **17(AUST-15 & Clone C)**, 318, 322, 380, 636, 688, 845, 958, 1255, 1313 | 10 | 9/10 = SLVs |
| CGTCGGCTATCCTTCCGGTG | 343, 381, 1256 | 3 | 3/3 = SLVs |
| CGTCGGCTATCCTTCGGGTA | 1163 | 1 |  |
| CGTCGGCTATCCTTTGGGTA | **497(Dutch-2)**, 544, 895, 1317 | 4 | 4/4 = SLVs or DLVs |
| CGTCGGCTCCCCCCCCGGCG | 168 | 1 |  |
| CGTCGGCTCCCCCCCCGGTA | 145, 1246 | 2 |  |
| CGTCGGCTCCCCCCCCGGTG | 485, 601, 855 | 3 | 2/3 = SLVs |
| CGTCGGCTCCCCCCCGAGCA | 473 | 1 |  |
| CGTCGGCTCCCCCCCGAGTG | 39 | 1 |  |
| CGTCGGCTCCCCCCCGGGTA | 752 | 1 |  |
| CGTCGGCTCCCCCCCGGGTG | 31, 690, 854 | 3 |  |
| CGTCGGCTCCCCCCTGGGTA | 194 | 1 |  |
| CGTCGGCTCCCCCCTGGGTG | 929 | 1 |  |
| CGTCGGCTCCCCTCCCGGCG | 149 | 1 |  |
| CGTCGGCTCCCCTCCGAGTG | 45, 52 | 2 | 2/2 = SLVs |
| CGTCGGCTCCCCTCTGGATG | 1325 | 1 |  |
| CGTCGGCTCCCCTCTGGGCA | 640 | 1 |  |
| CGTCGGCTCCCCTTCCGGCG | 1130 | 1 |  |
| CGTCGGCTCCCCTTCCGGTA | 557 | 1 |  |
| CGTCGGCTCCCCTTCCGGTG | 570, 1228 | 2 | 2/2 = SLVs |
| CGTCGGCTCCCCTTCGGGTA | 1308 | 1 |  |
| CGTCGGCTCCCCTTTCGGTG | 897 | 1 |  |
| CGTCGGCTCCCCTTTGAGTA | 24, 1345 | 2 | 2/2 = SLVs |
| CGTCGGCTCCCCTTTGGGTG | 1236 | 1 |  |
| CGTCGGCTCCCTCCCCGGCG | 500 | 1 |  |
| CGTCGGCTCCCTCCCGAGTG | 1381 | 1 |  |
| CGTCGGCTCCCTCCTGAGTG | 499 | 1 |  |
| CGTCGGCTCCTCCCCCGGTA | 1271 | 1 |  |
| CGTCGGCTCCTCCCCGGGCG | 910 | 1 |  |
| CGTCGGCTCCTCCCCGGGTG | 1143 | 1 |  |
| CGTCGGCTCCTCCCTCGGTA | 614 | 1 |  |
| CGTCGGCTCCTCCCTGGGCG | 221 | 1 |  |
| CGTCGGCTCCTCTCCCGGTA | 275 | 1 |  |
| CGTCGGCTCCTCTCCGAGTG | 340 | 1 |  |
| CGTCGGCTCCTCTCTGAGTA | 293 | 1 |  |
| CGTCGGCTCCTCTTCGGGTG | 1118 | 1 |  |
| CGTCGGCTCTCCCCCCAGCA | 627 | 1 |  |
| CGTCGGCTCTCCCCCCGGCG | 487 | 1 |  |
| CGTCGGCTCTCCCCCCGGTG | 583, 850 | 2 | 2/2 = DLVs |
| CGTCGGCTCTCCCCTGGACA | **1394(PACS2)** | 1 |  |
| CGTCGGCTCTCCTCCCAGTG | 160, 184 | 2 | 2/2 = SLVs |
| CGTCGGCTCTCCTCCGAGTA | 1384 | 1 |  |
| CGTCGGCTCTCCTCTGAGTA | 559 | 1 |  |
| CGTCGGCTCTCCTCTGAGTG | 1064 | 1 |  |
| CGTCGGCTCTCCTCTGGGTA | 483 | 1 |  |
| CGTCGGCTCTCCTTCCAGTA | 150 | 1 |  |
| CGTCGGCTCTCCTTTGGGTA | 147 | 1 |  |
| CGTCGGCTCTCCTTTGGGTG | 646 | 1 |  |
| CGTCGGCTGCCTCCCCGGCG | 737 | 1 |  |
| CGTCGGCTTCCGCCCCGACA | 489 | 1 |  |
| CGTCGGGCACCTCCCCGGCA | 1011 | 1 |  |
| CGTCGGGCACCTCCCGGGTA | 708 | 1 |  |
| CGTCGGGCACCTCCTGAGTG | 78 | 1 |  |
| CGTCGGGCACCTCCTGGGTA | 204 | 1 |  |
| CGTCGGGCACCTTCCCAGTA | 860 | 1 |  |
| CGTCGGGCACCTTCCCAGTG | 844 | 1 |  |
| CGTCGGGCACCTTCCCGGTA | 412 | 1 |  |
| CGTCGGGCACCTTCCGGGTA | 1164 | 1 |  |
| CGTCGGGCACCTTTCCGGTA | 59 | 1 |  |
| CGTCGGGCACTCCCCCAGTA | 1102 | 1 |  |
| CGTCGGGCACTCCCCCGACA | 851 | 1 |  |
| CGTCGGGCACTCCCCCGGTG | 60 | 1 |  |
| CGTCGGGCATCTTTCCAGTA | 422 | 1 |  |
| CGTCGGGCCCCCCCCGAGTG | 298, 446, 691, 1133, 1185 | 5 | 3/5 = SLVs or DLVs |
| CGTCGGGCCCCCCCTGAGTG | 255 | 1 |  |
| CGTCGGGCCCCCTCCCGGTA | 1083 | 1 |  |
| CGTCGGGCCCCCTCCGAGTG | 249, 921, 1047 | 3 | 2/3 = DLVs |
| CGTCGGGCCCCCTCCGGACG | 1371 | 1 |  |
| CGTCGGGCCCCCTCCGGGTG | 23 | 1 |  |
| CGTCGGGCCCCCTTCGGGTA | 32 | 1 |  |
| CGTCGGGCCCTCCCCGAGCA | 1023 | 1 |  |
| CGTCGGGCCCTCCCCGAGTG | 324 | 1 |  |
| CGTCGGGCCCTCTCCGAGTG | 1149, 1248 | 2 | 2/2 = SLVs |
| CGTCGGGCCTCCCCCCGACA | 852 | 1 |  |
| CGTCGGGCCTCCTCCGAGTA | 941 | 1 |  |
| CGTCGGGCCTCCTCTGAGTG | 909 | 1 |  |
| CGTCGGGTACCTCCTGAGTG | 1211 | 1 |  |
| CGTCGGGTACCTTCCCGGTA | 509 | 1 |  |
| CGTCGGGTATCCCCTGAGTA | 558 | 1 |  |
| CGTCGGGTATCCTTCCGGTA | 438 | 1 |  |
| CGTCGGGTCCCCCCCCGGTG | 1033 | 1 |  |
| CGTCGGGTCCCCCCCGAGTG | 539, 1216 | 2 | 2/2 = SLVs |
| CGTCGGGTCCCCTTCCGGCG | 1026 | 1 |  |
| CGTCGGGTCTCCCCCCAGTG | 770 | 1 |  |
| CGTCGGGTCTCCTCCCAGTG | 798 | 1 |  |
| CGTCGGGTCTCCTCCCGGCG | 736 | 1 |  |
| GATCGGGCCCCCTCCGAGTG | 317 | 1 |  |
| GGTCGGGCCCCCTCCGAGTG | 320 | 1 |  |
| TACCAGCCCCCCTTTGAGTG | 56 | 1 |  |
| TACCAGCTACCCTTCCAGTA | 139 | 1 |  |
| TACCAGCTACCCTTCCGGCA | 454, 1401 | 2 |  |
| TACCAGCTCCCCCCCGGGTA | 336 | 1 |  |
| TACCAGCTCCCCTCCGAGTG | 1027 | 1 |  |
| TACCAGCTCCTCCCCGAGTA | 82 | 1 |  |
| TACCAGCTCCTCCCCGAGTG | 1366 | 1 |  |
| TACCAGCTCCTCCCTGAGTG | 68 | 1 |  |
| TACCAGGCACCCTCCCGGTG | 273, 695 | 2 | 2/2 = SLVs |
| TACCAGGCCCCCCCCGAGTG | 631, 1019, 1284 | 3 | 2/3 = SLVs |
| TACCAGGCCCCCTCCGAGTA | 978 | 1 |  |
| TACCAGGCCCCCTCCGAGTG | 89, 307, **308(AUST-24)**, 662, 1028 | 5 | 2/5 = DLVs |
| TACCAGGCCCTCCCCCGGCG | 985 | 1 |  |
| TACCAGGCCCTCCCCGAGTG | 61, 223, 309, 311, 316, 325, 361, 383, 458, 1251, 1310 | 11 | 7/11 = SLVs or DLVs |
| TACCAGGCCCTCCCCGGGTG | 1372 | 1 |  |
| TACCAGGCCCTCCCTGAGTG | 757, 1022 | 2 |  |
| TACCAGGCCCTCTCCGAGTG | 658, 823, 1017, 1142 | 4 | 2/4 = SLVs |
| TACCAGGTACCCTCCCGGTG | 510 | 1 |  |
| TACCAGGTCCCCCCCGAGTG | 1187, 1224 | 2 |  |
| TACCAGGTCCCCCCTGAGTG | 1360 | 1 |  |
| TACCAGGTCCCCTCCGAGTG | 481, 1279 | 2 | 2/2 = DLVs |
| TACCAGGTCCTCCCCGAGTG | 507, 613, 1219 | 3 | 3/3 = SLVs |
| TACCAGGTCCTCCCCGGGTG | 80 | 1 |  |
| TACCAGTTACCCTTCCGGCA | 257 | 1 |  |
| TACCAGTTACCCTTCGGGCA | 780 | 1 |  |
| TACCGGGCCCTCTCCGAGCG | 999 | 1 |  |
| TATCGGCCACCTCCTGGGTA | 817 | 1 |  |
| TATCGGCCATCCCCCGAGTG | 995 | 1 |  |
| TATCGGCCCCTCCCCGAGCG | 927 | 1 |  |
| TATCGGCCCCTCCCCGAGTG | 205, 341 | 2 |  |
| TATCGGCCCCTCCCCGGGTG | 346 | 1 |  |
| TATCGGCCCCTCTCCGAGCG | 136 | 1 |  |
| TATCGGCCCCTCTCCGAGTG | 1259, 1260, 1261, 1262, 1334 | 5 | 5/5 = SLVs or DLVs |
| TATCGGCCGCTCCCCGAGTG | 667 | 1 |  |
| TATCGGCCGCTCCCCGGGTG | 468 | 1 |  |
| TATCGGCTACCTCCTGGGTA | 331 | 1 |  |
| TATCGGCTATCCCCCCGGTA | 329 | 1 |  |
| TATCGGCTATCCCCCGAGTG | 1368 | 1 |  |
| TATCGGCTATCCCCCGGGTG | 74 | 1 |  |
| TATCGGCTATCCTCCCGGTA | 333 | 1 |  |
| TATCGGCTATCCTCCGGGTG | 632 | 1 |  |
| TATCGGCTATCCTTCCGGTA | 1132 | 1 |  |
| TATCGGCTCCCCCCCGAGTG | 46 | 1 |  |
| TATCGGCTCCCCCCCGGGTG | 38 | 1 |  |
| TATCGGCTCCCCCCTGAGTG | 34 | 1 |  |
| TATCGGCTCCCCTCCGAGTG | 58, 1152, 1288 | 3 |  |
| TATCGGCTCCCCTCCGGACG | 126 | 1 |  |
| TATCGGCTCCCCTTCGAGTA | 40 | 1 |  |
| TATCGGCTCCCTCCCGGGTA | 300 | 1 |  |
| TATCGGCTCCTCCCCGAGCG | 588, 1323 | 2 | 2/2 = SLVs |
| TATCGGCTCCTCCCCGAGTG | 1267 | 1 |  |
| TATCGGCTCCTCCCCGGGCG | 582 | 1 |  |
| TATCGGCTCCTCTCCCAGTA | 66 | 1 |  |
| TATCGGCTCCTCTCCGAGCG | 518 | 1 |  |
| TATCGGCTCCTCTCCGAGTG | 55, 73, 560, 671, 729, 762, 1056, 1268 | 8 | 5/8 = SLVs or DLVs |
| TATCGGCTCCTCTCCGGGCA | 821 | 1 |  |
| TATCGGCTCCTCTCTGAGTG | 1263 | 1 |  |
| TATCGGCTCCTCTCTGGGCG | 982 | 1 |  |
| TATCGGCTCTCCTTCGAGTA | 44 | 1 |  |
| TATCGGCTGCTCCCCGAGTG | 502 | 1 |  |
| TATCGGGCACCTCCCGAGCG | 1116 | 1 |  |
| TATCGGGCACCTTCCGAGTG | 1282 | 1 |  |
| TATCGGGCACTCCCCCGGTA | 100 | 1 |  |
| TATCGGGCATCCTCCGGATG | 744 | 1 |  |
| TATCGGGCCCCCCCCCGGTA | 1379 | 1 |  |
| TATCGGGCCCCCCCCGAGCG | 319, 1160, 1400 | 3 | 2/3 = SLVs |
| TATCGGGCCCCCCCCGAGTA | 104 | 1 |  |
| TATCGGGCCCCCCCCGAGTG | 296, 306, 310, 694, 1197 | 5 | 4/5 = SLVs |
| TATCGGGCCCCCCCCGGGCG | 72 | 1 |  |
| TATCGGGCCCCCCCCGGGTG | 70, 237, 238, 243, 1001, 1158 | 6 | 4/6 = SLVs |
| TATCGGGCCCCCTCCCAGTG | 828 | 1 |  |
| TATCGGGCCCCCTCCGAGTG | 65, 107, 109, **253(PA14)**, 297, 338, 342, 377, 532, 773, 815, 923, 1110, 1363 | 14 | 3/14 = SLVs or DLVs |
| TATCGGGCCCCCTCCGGGTG | 85 | 1 |  |
| TATCGGGCCCCCTCTGAGTA | 1353 | 1 |  |
| TATCGGGCCCTCCCCGAGCG | 1078, 1182 | 2 | 2/2 = SLVs |
| TATCGGGCCCTCCCCGAGTG | 216, 246, 1072, 1192, 1203, 1206, 1329 | 7 |  |
| TATCGGGCCCTCCCCGGGTG | 279, 312, 1020, 1071, 1253 | 5 | 4/5 = SLVs or DLVs |
| TATCGGGCCCTCTCCCGGTG | 1383 | 1 |  |
| TATCGGGCCCTCTCCGAGCG | 830, 1076, 1112 | 3 |  |
| TATCGGGCCCTCTCCGAGTG | 63, 315, 701, 759, 816, 829, 926, 1093, 1138, 1146, 1254 | 11 | 3/11 = SLVs |
| TATCGGGCCCTCTCCGGGCG | 328, 1362 | 2 |  |
| TATCGGGCCCTCTCCGGGTG | 436, 930, 1042 | 3 |  |
| TATCGGGCCCTCTTCGAGTG | 1277 | 1 |  |
| TATCGGGCCTCCTCTGAGTA | 67 | 1 |  |
| TATCGGGTCCCCCCCGAGCG | 486 | 1 |  |
| TATCGGGTCCCCCCCGAGTG | 538 | 1 |  |
| TATCGGGTCCCCTCCGAGTG | 540, 551, 692, 1175 | 4 | 3/4 = SLVs |
| TATCGGGTCCCCTCTGAGTA | 42 | 1 |  |
| TATCGGGTCCCCTTCCAGTG | 967 | 1 |  |
| TATCGGGTCCTCCCCGAGCG | 537 | 1 |  |
| TATCGGGTCCTCTCCGAGTG | 493, 616, 1299 | 3 |  |
| TATCGGTTCCTCCCCCGGTA | 87 | 1 |  |
| TATCGGTTCCTCTCCCGGTA | 1373 | 1 |  |
| TGCAAACTCCCCTTTGGGCG | 456 | 1 |  |
| TGCAAGCCACCCCCTGGACA | 811 | 1 |  |
| TGCAAGCCACCTCCCCGACG | 417 | 1 |  |
| TGCAAGCCACCTCCCGGGCG | 117 | 1 |  |
| TGCAAGCCACCTTTTGAGTG | 1327 | 1 |  |
| TGCAAGCCCCCTTTTGAGTG | 624 | 1 |  |
| TGCAAGCTACCCCCCCGGCA | 7 | 1 |  |
| TGCAAGCTACCCCCCCGGCG | 1073 | 1 |  |
| TGCAAGCTACCCCCCGGACA | 250, 1341 | 2 | 2/2 = SLVs |
| TGCAAGCTACCCCCTGAGCA | 1010 | 1 |  |
| TGCAAGCTACCCCCTGGACA | 13, **155(AUST-10,-14, -19,&-37)**, 280, 541, 579, 677, **786(AUST-19)**, 1276, 1316, 1335 | 10 | 10/10 = SLVs |
| TGCAAGCTACCCCCTGGACG | 461 | 1 |  |
| TGCAAGCTACCCCCTGGGCA | 776 | 1 |  |
| TGCAAGCTACCCTCCCGGTA | 669, **808(AUST-21)** | 2 | 2/2 = SLVs |
| TGCAAGCTACCCTTCCAGTA | 115 | 1 |  |
| TGCAAGCTACCTCCCCGACG | **217(Manchester)**, 1134 | 2 | 2/2 = SLVs |
| TGCAAGCTACCTCCCCGGTG | 181 | 1 |  |
| TGCAAGCTACCTCCCGAGTA | 114 | 1 |  |
| TGCAAGCTACCTCCTGAGTG | 15 | 1 |  |
| TGCAAGCTACCTCCTGGACG | 14 | 1 |  |
| TGCAAGCTACCTCTTGGGTG | 128, 450, 676 | 3 | 3/3 = SLVs |
| TGCAAGCTACCTTCCCGGCG | 684 | 1 |  |
| TGCAAGCTACCTTCCCGGTA | **775(AUST-02)** | 1 |  |
| TGCAAGCTACCTTCCGGGTA | 807 | 1 |  |
| TGCAAGCTACCTTCTGAGTA | 763 | 1 |  |
| TGCAAGCTACCTTCTGAGTG | 428, 1196 | 2 |  |
| TGCAAGCTACCTTCTGGGTA | 778 | 1 |  |
| TGCAAGCTACCTTCTGGGTG | 933 | 1 |  |
| TGCAAGCTACCTTTCCAGTA | 1029 | 1 |  |
| TGCAAGCTACCTTTCCGGCA | 1229 | 1 |  |
| TGCAAGCTACCTTTCCGGCG | 151 | 1 |  |
| TGCAAGCTACCTTTCCGGTG | 387, 404 | 2 | 2/2 = DLVs |
| TGCAAGCTACCTTTCGAGTG | 1240 | 1 |  |
| TGCAAGCTACCTTTCGGGTG | 730 | 1 |  |
| TGCAAGCTACCTTTTCAGTA | 552 | 1 |  |
| TGCAAGCTACCTTTTGGACA | 11 | 1 |  |
| TGCAAGCTATCCCCCCGGTG | **12(AUST-33)** | 1 |  |
| TGCAAGCTATCCCCCGAGTA | 1275 | 1 |  |
| TGCAAGCTATCCCCCGGGCA | 526 | 1 |  |
| TGCAAGCTATCCCCTCGGTA | 1399 | 1 |  |
| TGCAAGCTATCCCCTGAGTA | 1031 | 1 |  |
| TGCAAGCTATCCCCTGGGTA | 1330 | 1 |  |
| TGCAAGCTATCCTCCCAGCG | **236(AUST-32)**, 239, 240 | 3 | 3/3 = SLVs |
| TGCAAGCTATCCTCCCGGCG | 8 | 1 |  |
| TGCAAGCTATCCTCTGAGTA | 521 | 1 |  |
| TGCAAGCTATCCTTTGGACG | 1040 | 1 |  |
| TGCAAGCTCACGCCCCAGTA | 548 | 1 |  |
| TGCAAGCTCCCCCCCCAGCA | 643 | 1 |  |
| TGCAAGCTCCCCCCCCGGTG | 572 | 1 |  |
| TGCAAGCTCCCCCCCGAGTG | 1250 | 1 |  |
| TGCAAGCTCCCCCCCGGGTA | 907, 990 | 2 | 2/2 = DLVs |
| TGCAAGCTCCCCCCTGGGTG | 171 | 1 |  |
| TGCAAGCTCCCCTCCGAGTG | 793 | 1 |  |
| TGCAAGCTCCCCTCTGGGCG | 735 | 1 |  |
| TGCAAGCTCCCCTTCCGGCA | 25 | 1 |  |
| TGCAAGCTCCCCTTCCGGTA | 1053 | 1 |  |
| TGCAAGCTCCCCTTCGGGTA | 267, 440 | 2 | 2/2 = SLVs |
| TGCAAGCTCCCCTTTCAGCG | 16 | 1 |  |
| TGCAAGCTCCCCTTTGGGCG | **242(AUST-03)**, 996 | 2 | 2/2 = SLVs |
| TGCAAGCTCCCTTCCCGGTA | 914 | 1 |  |
| TGCAAGCTCCTCCCCCGGTA | 33, 993, 1350 | 3 | 2/3 = SLVs |
| TGCAAGCTCCTCCCCCGGTG | 1289 | 1 |  |
| TGCAAGCTCCTCTCCGAGTG | 332 | 1 |  |
| TGCAAGCTCCTCTCTGAGCA | 571, 1012 | 2 | 2/2 = SLVs |
| TGCAAGCTCCTCTCTGGACA | 1013 | 1 |  |
| TGCAAGCTCCTCTTCCGGCG | 612 | 1 |  |
| TGCAAGCTCTCCCCCCGGTG | 1397 | 1 |  |
| TGCAAGCTCTCCTCTGAGTG | 362 | 1 |  |
| TGCAAGCTCTCCTCTGGGTG | 355, 356 | 2 | 2/2 = SLVs |
| TGCAAGCTCTCCTTCCGGTA | 41 | 1 |  |
| TGCAAGCTCTCCTTCCGGTG | 153 | 1 |  |
| TGCAAGCTCTCCTTCGAGCG | 906 | 1 |  |
| TGCAAGGCACCTTTCCGGCA | 64 | 1 |  |
| TGCAAGGCACTCCCCCGGTG | 1361 | 1 |  |
| TGCAAGGCCCCCCCCCAGTA | 972 | 1 |  |
| TGCAAGGCCCCCCCCCGGTA | 177 | 1 |  |
| TGCAAGGCCCCCCCCCGGTG | 176 | 1 |  |
| TGCAAGGCCCCCCCCGAGTG | 69, 1367 | 2 | 2/2 = SLVs |
| TGCAAGGCCCCCCCCGGGTG | 263 | 1 |  |
| TGCAAGGCCCCCCCTGGGTA | 71 | 1 |  |
| TGCAAGGCCCCCTCCGAGTG | 753 | 1 |  |
| TGCAAGGCCCCCTCCGGGTG | 644, 935 | 2 |  |
| TGCAAGGCCCCCTTTGGGTG | 1014 | 1 |  |
| TGCAAGGCCCTCTTCCAGTG | 1209 | 1 |  |
| TGCAAGGTACCTTTCCGACG | 437 | 1 |  |
| TGCAAGGTCCCCCCCGGGTG | 228 | 1 |  |
| TGCAAGGTCCCCCCTGGGTG | 159, 175, 619 | 3 | 3/3 = SLVs or DLVs |
| TGCAAGGTCCCCTCCGGGTA | 777 | 1 |  |
| TGCAAGGTCCCCTCTGGGTG | 201 | 1 |  |
| TGCAAGGTCCTCCCTGGGTG | 1139 | 1 |  |
| TGCAAGTTACCCTCCCAGTA | 542 | 1 |  |
| TGCAAGTTACCCTCCGAGTA | 1194 | 1 |  |
| TGCAAGTTACCCTCCGAGTG | 459 | 1 |  |
| TGCAAGTTACCTCCTGGGCA | 116 | 1 |  |
| TGCAGGCCCCTCCCCGGGTG | 704 | 1 |  |
| TGCAGGCTACCCTTTCGGTA | 1176 | 1 |  |
| TGCAGGCTCCCCCCCCAGCA | **549(PA01)**, 1331 | 2 | 2/2 = SLVs |
| TGCAGGCTCCCTCCCCAGCA | 699 | 1 |  |
| TGCAGGCTCCCTCCCCAGCG | 764 | 1 |  |
| TGCAGGCTCCCTCCCCAGTA | 944 | 1 |  |
| TGCAGGGCACCTTTCCAGTA | 305 | 1 |  |
| TGCAGGGCACTCCCTGAGTA | 1346 | 1 |  |
| TGCAGGGCCCCCCCTGAGTA | 29 | 1 |  |
| TGCAGGGCCCCCTCCGAGTG | 326 | 1 |  |
| TGCAGGGCCCCTTCTGAGTA | 95 | 1 |  |
| TGCAGGGTCCCCCCTGGGTG | 101 | 1 |  |
| TGCCAACCCCCTCCCGAGCG | 678 | 1 |  |
| TGCCAGCTCCCCCCCGAGTG | 1092 | 1 |  |
| TGCCAGCTCCCCTCCGAGTG | 550 | 1 |  |
| TGCCAGCTCCTCCCCGAGTG | 383, 765, 1339 | 3 | 2/3 = SLVs |
| TGCCAGGCACCCCCCCGGCA | 1099, 1100 | 2 | 2/2 = SLVs |
| TGCCAGGCCCCCCCCGAGTG | 1141, 1150, 1257, 1258 | 4 | 3/4 = SLVs |
| TGCCAGGCCCCCTCCGAGTG | 207 | 1 |  |
| TGCCAGGCCCCTCCCGAGCG | 313, 648 | 2 | 2/2 = SLVs |
| TGCCAGGCCCCTCCCGGGTG | 174 | 1 |  |
| TGCCAGGCCCTCCCCGAGTG | 1121 | 1 |  |
| TGCCAGGCCCTCTCCCAGTG | 352 | 1 |  |
| TGCCAGGCCCTCTCCGAGTG | 1051 | 1 |  |
| TGCCAGGTCCCCCCCGAGTG | 565 | 1 |  |
| TGCCAGGTCCCCTCCGAGTG | 1287 | 1 |  |
| TGCCGGCCATCCCCCCGGCA | 423 | 1 |  |
| TGCCGGCCCCCCTTCGGGCG | 1109 | 1 |  |
| TGCCGGCCCCTCCCCCAGTA | 391 | 1 |  |
| TGCCGGCCCCTCCCCGGGCG | 728 | 1 |  |
| TGCCGGCTACCCCCCCAGCG | 876 | 1 |  |
| TGCCGGCTACCCCCCCAGTG | 720, 988 | 2 |  |
| TGCCGGCTACCCCCCCGACA | 747, 875 | 2 |  |
| TGCCGGCTACCCCCCGAGTA | 1280 | 1 |  |
| TGCCGGCTACCTCCCCAGTG | 617, 706 | 2 |  |
| TGCCGGCTACCTCCCCGGTG | 81, 980, 1117 | 3 | 2/3 = DLVs |
| TGCCGGCTACCTCCCGAGTA | 873 | 1 |  |
| TGCCGGCTACCTCCCGGGCA | 805 | 1 |  |
| TGCCGGCTACCTCCTGAGTA | 266 | 1 |  |
| TGCCGGCTACCTTCCCAGTA | 488 | 1 |  |
| TGCCGGCTACCTTCCCGGTG | 1169 | 1 |  |
| TGCCGGCTACCTTCTGAGTA | 1269 | 1 |  |
| TGCCGGCTACCTTTCCGGTA | 393 | 1 |  |
| TGCCGGCTACCTTTCGAGTG | 453 | 1 |  |
| TGCCGGCTACCTTTCGGGTA | 634, 1291 | 2 | 2/2 = DLVs |
| TGCCGGCTACCTTTTCAGTA | 433 | 1 |  |
| TGCCGGCTACTCTTCCGACA | 874 | 1 |  |
| TGCCGGCTATCCCCCCAGTA | 899 | 1 |  |
| TGCCGGCTATCCCCCCGACA | 787, **788(AUST-04)** | 2 | 2/2 = SLVs |
| TGCCGGCTATCCCCCCGGCA | **822(AUST-11)**, **1239(M18)** | 2 | 2/2 = SLVs |
| TGCCGGCTATCCCCCCGGTA | 879 | 1 |  |
| TGCCGGCTATCCCCTGAGTA | 173 | 1 |  |
| TGCCGGCTATCCTCTGAGTG | 1293 | 1 |  |
| TGCCGGCTATCCTTCCGATA | 233, 375 | 2 | 2/2 = DLVs |
| TGCCGGCTATCCTTCCGGCG | 193 | 1 |  |
| TGCCGGCTATCCTTCCGGTA | 742 | 1 |  |
| TGCCGGCTATCCTTCCGGTG | 1294 | 1 |  |
| TGCCGGCTATCCTTTGAGTA | 555, 573 | 2 | 2/2 = SLVs |
| TGCCGGCTATCCTTTGGATA | 1391 | 1 |  |
| TGCCGGCTCCCCCCCCGACA | 156 | 1 |  |
| TGCCGGCTCCCCCCCGAGTG | 1220 | 1 |  |
| TGCCGGCTCCCCCCCGGACA | 1386 | 1 |  |
| TGCCGGCTCCCCCCTGAGCA | 1148, 1396 | 2 | 2/2 = SLVs |
| TGCCGGCTCCCCCCTGGGCA | 554, **804(AUST-11)** | 2 | 2/2 = SLVs |
| TGCCGGCTCCCCTCTGAGCG | 62 | 1 |  |
| TGCCGGCTCCCCTTCCGGTG | 54, 1358 | 2 | 2/2 = SLVs |
| TGCCGGCTCCCCTTCGAGTG | 21 | 1 |  |
| TGCCGGCTCCCCTTTGGGCG | 350, 354, 525, 603, 1321 | 5 | 5/5 = SLVs or DLVs |
| TGCCGGCTCCCCTTTGGGTA | 303 | 1 |  |
| TGCCGGCTCCCCTTTGGGTG | 1336 | 1 |  |
| TGCCGGCTCCCTCCCGGGCA | 806 | 1 |  |
| TGCCGGCTCCCTCCCGGGTA | 491 | 1 |  |
| TGCCGGCTCCTCCCCGGGCA | 716 | 1 |  |
| TGCCGGCTCCTCTCCCAGTA | 1364 | 1 |  |
| TGCCGGGCACCTCCCCGGCG | 475 | 1 |  |
| TGCCGGGCACCTTTCCGGCG | 1101 | 1 |  |
| TGCCGGGCATCCCCTGGACG | 26 | 1 |  |
| TGCCGGGCATCCTCCGAATG | 871 | 1 |  |
| TGCCGGGCATCCTCCGGACG | 870 | 1 |  |
| TGCCGGGCCCCCCCCGAGTG | 357, 746, 1153 | 3 | 3/3 = SLVs |
| TGCCGGGCCCCCCCCGGGCA | 1085 | 1 |  |
| TGCCGGGCCCCCTCTGAGTG | 1281 | 1 |  |
| TGCCGGGCCCTCCCCCGGTG | 872 | 1 |  |
| TGCCGGGCCCTCCCCGAGTG | 1024 | 1 |  |
| TGCCGGGCCCTCCCCGGGCG | 755 | 1 |  |
| TGCCGGGTACCCTCCCAGTG | 748 | 1 |  |
| TGCCGGGTACCTCCCCGGCA | 878 | 1 |  |
| TGCCGGGTACCTCCCCGGCG | 527 | 1 |  |
| TGCCGGGTACCTCCCCGGTG | 992 | 1 |  |
| TGCCGGGTATCCTCCGAATG | 596 | 1 |  |
| TGCCGGGTATCCTTCCGATA | 743 | 1 |  |
| TGCCGGGTATCCTTTGGGCG | 900 | 1 |  |
| TGCCGGGTCCCCCCTGGGCG | 1094, 1265 | 2 | 2/2 = SLVs |
| TGCCGGGTCCCCCTTGGGCG | 877 | 1 |  |
| TGCCGGGTCCCCTTCCAGTG | 609 | 1 |  |
| TGCCGGGTCTCCTTTGGACA | 285 | 1 |  |
| TGCTAGGCCCCCTCCGAGTG | 304, 323 | 2 | 2/2 = SLVs |
| TGTAAGCTACCTTCTGGGCG | 290 | 1 |  |
| TGTAAGCTCCCCTTCCGGCA | 199 | 1 |  |
| TGTAAGGCCCCCTCCGAGTG | 628 | 1 |  |
| TGTCGACTACCTCCCCGGCA | 1008 | 1 |  |
| TGTCGACTCCTCTTCCGGTA | 439, 515 | 2 | 2/2 = SLVs |
| TGTCGCCTCCCCCCCGCGTC | 721 | 1 |  |
| TGTCGGCCACCCCCCCAGCA | 718 | 1 |  |
| TGTCGGCCACCCCCCGGGCG | 19 | 1 |  |
| TGTCGGCCACCTCCCCGGTG | 414 | 1 |  |
| TGTCGGCCACCTCCTCGGTG | 351 | 1 |  |
| TGTCGGCCACCTTCCGAGTA | 214 | 1 |  |
| TGTCGGCCACCTTCTGAGTG | 431 | 1 |  |
| TGTCGGCCACCTTTCGAGTA | 917 | 1 |  |
| TGTCGGCCACCTTTTGGGTG | 410 | 1 |  |
| TGTCGGCCATCCCCCCGGTA | 2 | 1 |  |
| TGTCGGCCATCCCCTGGGTA | 1074 | 1 |  |
| TGTCGGCCATCCTCCGGGTG | 625 | 1 |  |
| TGTCGGCCATCCTCTCGGTA | 470 | 1 |  |
| TGTCGGCCCCCCCCCGGGTA | 118 | 1 |  |
| TGTCGGCCCCCCCCTGGGTA | 9,163 | 2 | 2/2 = SLVs |
| TGTCGGCCCCCCTCTGGGTA | 154 | 1 |  |
| TGTCGGCCCCCCTTCCGGTG | 434 | 1 |  |
| TGTCGGCCCCCCTTCGGGTA | **455(AUST-27)** | 1 |  |
| TGTCGGCCCCCTCCCCGGTG | 418 | 1 |  |
| TGTCGGCCCCCTCCCGGGTG | 472 | 1 |  |
| TGTCGGCCCCTCCCCGAGTG | 712, 756 | 2 | 2/2 = SLVs |
| TGTCGGCCCTCCCCCCGGCG | 925 | 1 |  |
| TGTCGGCCCTCCTCCCGGTA | 421 | 1 |  |
| TGTCGGCCCTCCTCCGGACA | 1018 | 1 |  |
| TGTCGGCCCTCCTCTCGGTA | 477 | 1 |  |
| TGTCGGCTACCCCCCCGGCA | 231, 370, 376 | 3 | 3/3 = SLVs |
| TGTCGGCTACCCCCCCGGCG | 1264 | 1 |  |
| TGTCGGCTACCCCCCCGGTG | 213 | 1 |  |
| TGTCGGCTACCCCCCGAGTG | 330 | 1 |  |
| TGTCGGCTACCCCCCGGGCG | 1343 | 1 |  |
| TGTCGGCTACCCCCTGGGCA | 211, 642, 1184 | 3 | 3/3 = SLVs or DLVs |
| TGTCGGCTACCCCCTGGGTA | 420, 924 | 2 | 2/2 = SLVs |
| TGTCGGCTACCCTCCCGGCG | 196 | 1 |  |
| TGTCGGCTACCCTCCGGGTG | 442 | 1 |  |
| TGTCGGCTACCCTCTGGGTG | 1030 | 1 |  |
| TGTCGGCTACCCTTCCAGTA | 567, 587 | 2 | 2/2 = DLVs |
| TGTCGGCTACCCTTCCGACA | 260, 264, 503 | 3 | 3/3 = SLVs |
| TGTCGGCTACCCTTCGGGCG | 289, 1298 | 2 |  |
| TGTCGGCTACCCTTTGGGTA | 1044 | 1 |  |
| TGTCGGCTACCTCCCCAGCG | 1205 | 1 |  |
| TGTCGGCTACCTCCCCAGTA | 664, 1167, 1337 | 3 | 2/3 = SLVs |
| TGTCGGCTACCTCCCCAGTG | 680 | 1 |  |
| TGTCGGCTACCTCCCCGGTA | 269, 686, 687 | 3 | 3/3 = SLVs |
| TGTCGGCTACCTCCCCGGTG | **146(LES)**, 374, 467, 681, 683, 970 | 6 | 6/6 = SLVs |
| TGTCGGCTACCTCCCGAGTG | 635 | 1 |  |
| TGTCGGCTACCTCCCGGGTG | 768, 1180 | 2 |  |
| TGTCGGCTACCTCCTGAGTG | 779 | 1 |  |
| TGTCGGCTACCTCCTGGGCA | 186 | 1 |  |
| TGTCGGCTACCTCCTGGGCG | 792, 1340 | 2 | 2/2 = SLVs |
| TGTCGGCTACCTTCCCAGTA | 834 | 1 |  |
| TGTCGGCTACCTTCCCAGTG | 1096 | 1 |  |
| TGTCGGCTACCTTCCCGGTA | **833(AUST-23)**, 839 | 2 | 2/2 = SLVs |
| TGTCGGCTACCTTCTGAGTG | 1091 | 1 |  |
| TGTCGGCTACCTTCTGGGTA | 1202 | 1 |  |
| TGTCGGCTACCTTTCCAGCG | 591, 761, 1049 | 3 | 3/3 = SLVs or DLVs |
| TGTCGGCTACCTTTCGAGTA | 1303 | 1 |  |
| TGTCGGCTACCTTTCGGGCG | 131 | 1 |  |
| TGTCGGCTACCTTTCGGGTA | 209, 268, **274(AUST-05,-09, -18,-25,&-31)** , 466, 546, **781(AUST-05)**, 936, **1043(AUST-09)**, 1068, 1089, 1301, 1326 | 12 | 12/12 = SLVs |
| TGTCGGCTACCTTTCGGGTG | 134 | 1 |  |
| TGTCGGCTACCTTTTGGGCG | 663, 969 | 2 |  |
| TGTCGGCTACCTTTTGGGTG | 282, 444, 959 | 3 | 3/3 = SLVs |
| TGTCGGCTACTCCCCCAGTA | 152, 185 | 2 | 2/2 = SLVs |
| TGTCGGCTACTCCCTGGACA | 994 | 1 |  |
| TGTCGGCTACTCCCTGGGTA | 543 | 1 |  |
| TGTCGGCTACTCTCCGGGTA | 1127 | 1 |  |
| TGTCGGCTACTCTCCGGGTG | 1204 | 1 |  |
| TGTCGGCTACTCTTTGGGTA | 208 | 1 |  |
| TGTCGGCTATCCCCCCAGTA | 413, 974 | 2 | 2/2 = SLVs |
| TGTCGGCTATCCCCCCAGTG | 286 | 1 |  |
| TGTCGGCTATCCCCCCGGTA | 132, 212, 607, 615, 665 | 5 | 4/5 = SLVs |
| TGTCGGCTATCCCCCCGGTG | 1090 | 1 |  |
| TGTCGGCTATCCCCCGGGCG | 524 | 1 |  |
| TGTCGGCTATCCCCCGGGTG | 407, 661, 1283 | 3 | 2/3 = SLVs |
| TGTCGGCTATCCCCTGGACG | 837 | 1 |  |
| TGTCGGCTATCCCCTGGGCA | 656 | 1 |  |
| TGTCGGCTATCCCCTGGGTA | 835 | 1 |  |
| TGTCGGCTATCCCCTGGGTG | 731, 838, 1052 | 3 | 3/3 = SLVs or DLVs |
| TGTCGGCTATCCTCCCAGTG | 1097 | 1 |  |
| TGTCGGCTATCCTCCCGGTA | 335 | 1 |  |
| TGTCGGCTATCCTCCGGGTA | 511, 1322 | 2 | 2/2 = SLVs |
| TGTCGGCTATCCTCCGGGTG | 112, 395, 841 | 3 | 2/3 = SLVs |
| TGTCGGCTATCCTCTCGGCA | 836 | 1 |  |
| TGTCGGCTATCCTCTGGGTA | 1016 | 1 |  |
| TGTCGGCTATCCTCTGGGTG | 932, 1115 | 2 |  |
| TGTCGGCTATCCTTCCAGTA | 182, 449 | 2 | 2/2 = SLVs |
| TGTCGGCTATCCTTCCGGTA | 157, 187, 321, 339, 505 | 5 | 4/5 = SLVs |
| TGTCGGCTATCCTTCGAGTA | 1377 | 1 |  |
| TGTCGGCTATCCTTCGGGCG | 585 | 1 |  |
| TGTCGGCTATCCTTTCAGTA | 96 | 1 |  |
| TGTCGGCTATCCTTTGAGTA | 295 | 1 |  |
| TGTCGGCTATCCTTTGGGCA | 668 | 1 |  |
| TGTCGGCTATCTTTTGGGTG | 278 | 1 |  |
| TGTCGGCTCCCCCCCCAGTG | 1232 | 1 |  |
| TGTCGGCTCCCCCCCCGACG | 915 | 1 |  |
| TGTCGGCTCCCCCCCCGGTA | 291, 1107 | 2 |  |
| TGTCGGCTCCCCCCCCGGTG | 842 | 1 |  |
| TGTCGGCTCCCCCCCGAGTG | 463 | 1 |  |
| TGTCGGCTCCCCCCCGGGTA | 105, 1296 | 2 |  |
| TGTCGGCTCCCCCCTCGGTA | 1306 | 1 |  |
| TGTCGGCTCCCCCCTGAGTA | 1285 | 1 |  |
| TGTCGGCTCCCCCCTGAGTG | 968 | 1 |  |
| TGTCGGCTCCCCCCTGGACG | 256, 645, 1025, 1333 | 4 | 4/4 = SLVs |
| TGTCGGCTCCCCCCTGGGTA | 252, 411, 429, 495, 702, 984 | 6 | 6/6 = SLVs |
| TGTCGGCTCCCCTCCCGACA | 1270 | 1 |  |
| TGTCGGCTCCCCTCCGGGTA | 1105 | 1 |  |
| TGTCGGCTCCCCTCCGGGTG | 48, 795, 1354 | 3 | 2/3 = SLVs |
| TGTCGGCTCCCCTCTGGGTA | 531 | 1 |  |
| TGTCGGCTCCCCTTCCGGTG | 840 | 1 |  |
| TGTCGGCTCCCCTTCGGGTA | 578 | 1 |  |
| TGTCGGCTCCCCTTTGGGCA | 564, 604, 1188 | 3 | 3/3 = SLVs or DLVs |
| TGTCGGCTCCCTCCCCGGTG | 415 | 1 |  |
| TGTCGGCTCCCTCCCGAGTG | 122 | 1 |  |
| TGTCGGCTCCCTTCCCGGTG | 135 | 1 |  |
| TGTCGGCTCCCTTCCGGGTG | 1210 | 1 |  |
| TGTCGGCTCCTCCCTGAGTG | 496 | 1 |  |
| TGTCGGCTCCTCCCTGGGTA | 498 | 1 |  |
| TGTCGGCTCCTCCCTGGGTG | 409 | 1 |  |
| TGTCGGCTCCTCTCCCAGTG | 975 | 1 |  |
| TGTCGGCTCCTCTCCGAGTG | 137 | 1 |  |
| TGTCGGCTCCTCTCCGGGTG | 606 | 1 |  |
| TGTCGGCTCCTCTCTGGGCG | 1060 | 1 |  |
| TGTCGGCTCCTCTTTGAGTA | 666, 769 | 2 |  |
| TGTCGGCTCCTCTTTGGGTA | **254(AUST-38**), **1041(AUST-38)** | 2 | 2/2 = SLVs |
| TGTCGGCTCTAACCCCAGTA | 749 | 1 |  |
| TGTCGGCTCTCCCCCCGACA | 1066 | 1 |  |
| TGTCGGCTCTCCCCCCGACG | 633 | 1 |  |
| TGTCGGCTCTCCCCCCGGCG | 1179 | 1 |  |
| TGTCGGCTCTCCCCTGGACA | 672 | 1 |  |
| TGTCGGCTCTCCTCCCAGTA | 1082 | 1 |  |
| TGTCGGCTCTCCTCCCGGTA | 641, 973, 1245 | 3 | 3/3 = SLVs |
| TGTCGGCTCTCCTCCGGGTA | 1166 | 1 |  |
| TGTCGGCTCTCCTCCGGGTG | 618 | 1 |  |
| TGTCGGCTCTCCTCTCGGTA | 162 | 1 |  |
| TGTCGGCTCTCCTTCCAGTA | 27, 294, 334, 1208, 1324 | 5 | 5/5 = SLVs or DLVs |
| TGTCGGCTCTCCTTTGAGTA | 119, 129, 1382 | 3 | 3/3 = SLVs |
| TGTCGGGCACCTCCCCAGTG | 37 | 1 |  |
| TGTCGGGCACCTCCCCGGCA | 1135 | 1 |  |
| TGTCGGGCACCTTCTGGGTA | 979 | 1 |  |
| TGTCGGGCACTCCCCCAGTA | 465 | 1 |  |
| TGTCGGGCACTCTTTGGGTA | 1108 | 1 |  |
| TGTCGGGCATCCCCCGAGTG | 943 | 1 |  |
| TGTCGGGCATCCCCCGGGTG | 832 | 1 |  |
| TGTCGGGCATCCCCTGGGTG | 190 | 1 |  |
| TGTCGGGCATCCTCCCGGTG | 84 | 1 |  |
| TGTCGGGCATCCTCTGAGTG | 314 | 1 |  |
| TGTCGGGCATCCTCTGGGTG | 713 | 1 |  |
| TGTCGGGCATCCTTCCAGTG | 18 | 1 |  |
| TGTCGGGCCCCCCCCGAGTG | 344, 650, 1131 | 3 | 3/3 = SLVs or DLVs |
| TGTCGGGCCCCCCCTGAGCA | 22 | 1 |  |
| TGTCGGGCCCCCCCTGAGTA | 991 | 1 |  |
| TGTCGGGCCCCCCCTGGGTG | 831 | 1 |  |
| TGTCGGGCCCCCTCCCAGTG | 771 | 1 |  |
| TGTCGGGCCCCCTCCGAGTG | 345, 620, 674 | 3 |  |
| TGTCGGGCCCTCCCCGGGCG | 751 | 1 |  |
| TGTCGGGCCCTCTCCGAGCG | 1249 | 1 |  |
| TGTCGGGCCCTCTCCGAGTG | 843 | 1 |  |
| TGTCGGGCCCTCTCCGGGTG | 1178, 1332 | 2 | 2/2 = SLVs |
| TGTCGGGCCTCCTCTGAGTA | 1365 | 1 |  |
| TGTCGGGTACCTTCTGGGTA | 50 | 1 |  |
| TGTCGGGTATCCCCTGGGTG | 566 | 1 |  |
| TGTCGGGTCCCCCCCGAGTG | 1286 | 1 |  |
| TGTCGGGTCCCCTCCGAGTG | 520 | 1 |  |
| TGTCGGGTCCTCTCCGAGCG | 1155 | 1 |  |
| TGTCGGGTCCTCTCCGAGTG | 3 | 1 |  |
| TGTCGGGTCCTCTCCGGGTG | 563 | 1 |  |
| TGTCGGGTCTCCTTCCAGTA | 120 | 1 |  |

^a^ SNP profile is in the order of 7, 45, 322, 381, 387, 416, 488, 881, 894, 937, 1086, 1152, 1297, 1465, 1865, 1958, 2169, 2208, 2337 and 2551. These SNPs were derived from the sequence data from the *Pseudomonas aeruginosa* MLST database website ([http://pubmlst.org/paeruginosa](http://pubmlst.org/pa)) on 13 December 2012.^b^ MLST types available from the *Pseudomonas aeruginosa* MLST database website ([http://pubmlst.org/paeruginosa](http://pubmlst.org/pa) ) on 13 December 2012. ^c^SLV: single locus variant, DLV: double locus variant.
