# Supplementary material for: A comparison of two informative SNP-based strategies for typing Pseudomonas aeruginosa isolates from patients with cystic fibrosis
Source: BMC Infect Dis. 2014 Jun 5;14:307. doi: 10.1186/1471-2334-14-307 (PMC4053291; doi:10.1186/1471-2334-14-307)
Supplement: Additional file 3: Table S3 — iPLEX20SNP and HRM10SNP results for the 506 isolates. iPLEX20SNP predicted STs or HRM10SNP profiles consistent with recognised P. aeruginosa strains are indicated in parentheses. [file 1471-2334-14-307-S3.docx]

**Table S3.** iPLEX20SNP and HRM10SNP results for the 506 isolates. iPLEX20SNP predicted STs or HRM10SNP profiles consistent with recognised *P. aeruginosa* strains are indicated in parentheses.

| iPLEX20SNP | iPLEX20SNP predicted MLST | HRM10SNP | No. of Isolates | Source |
| --- | --- | --- | --- | --- |
| CGCAAACTCTCCTTCCAGCA | 5 | CCTTCCAGCA | 1 | WA |
| CGCAAGCCCCTCTTTGGGCA | 740 | CCTCCCAGTA | 1 | WA |
| CGCAAGCTACCCCCTGAGTG | 299 | CCTCCTGGCG | 1 | VIC |
| CGCAAGCTACCCCCTGGGTG | 904 | CCTTCCGGCG | 1 | QLD |
| CGCAAGCTACCTCCCCAGTA | **882(AUST-11)**, 1151, 1233 | CCTCTCGGCA | 2 | QLD [1], WA[1] |
| CGCAAGCTACCTCCCCGGTA | 384, **1037(AUST-11)** | CCTCTCGGTA | 1 | WA |
| CGCAAGCTACCTCCTGGACG | NT1 | CCTCTCAGTG | 1 | SA |
| CGCAAGCTACCTTCCCGGTA | 232, **241(AUST-28)**, 247, 379, 471, 577 | CCTCTCGGTA | 4 | NSW [2], VIC [2] |
| CGCAAGCTACTCTCCCGGTG | **905(AUST-16)**, 1039 | CCTTCCGGTG | 11 | NSW [4], SA [6], VIC [1] |
| CGCAAGCTACTCTTCCGGCG | 902 | CCTCCCAGTG | 1 | QLD |
| CGCAAGCTATCCCCCCGGCA | **262(AUST-07)**, 774, 1165 | CCTCCCAGTA | 4 | NSW [1], QLD [2], VIC [1] |
| CGCAAGCTATCCCCCGGGTG ** | 4, **801(AUST-06)**, 1292 | CCTCCTGATG **(AUST-06)** | 11 | NSW [1], QLD [9], VIC [1] |
|  |  | CCTCCTGACG^1^ | 1 | QLD |
| CGCAAGCTATCCCCTGGACG | 962 | CCTCCCAGTG | 1 | NSW |
| CGCAAGCTATCCTTCGGGTA | NT2 | CCTCCCGGTA | 1 | NSW |
| CGCAAGCTCCCCCCCGGGTA | 103, 244, 441, 462, 464, 594, 766, 986, **1038(AUST-34)**, 1181, 1227,1338 | CCTTCTGGCA | 1 | VIC |
| CGCAAGCTCCTCTTTCGGTA | **277(AUST-36)**, 364, 1128, 1390 | CCTCCCGGTA | 1 | WA |
| CGCAAGGCATCCTTCCGGTA | 1009, 1067 | CCTTCCGATA | 1 | WA |
| CGCAAGGTACCTCCTGAGTG | 880 | CCTCTTGGTG | 1 | QLD |
| CGCAAGGTACCTCCTGGGCG | **800(AUST-13)** | CCTCTCAGTG | 2 | QLD [1], NSW [1] |
| CGCAAGGTCCCCCCCGGGTG | **389(AUST-13)** | CCTCCCGGTG | 2 | NSW |
| CGCAAGGTCCCCTTTGAGCG | 794 | CCTCCCAGTG | 2 | WA |
| CGCAAGTTCTCTTCTGAGTG | 697 | CCTCTCGGCG | 3 | QLD [1], WA [2] |
| CGCAGACCCCGCCCCGGGTG | 200,215,637,679,705,947, 955,1045,1198,1225,1328 | CCCCCTGGCG | 1 | WA |
| CGCAGGCCACCTTTCCGGTA | NT3 | CCCCTCGGTA | 1 | NSW |
| CGCAGGCCCCCCTTCGGGCG | NT4 | CCTCCCAGTG | 1 | NSW |
| CGCAGGCCCCTCCCCGGGTG | 195,224,349,359,719,961,977,1077,1183,1221,1222, 1266,1278 | CCCCCCGGCG | 1 | VIC |
| CGCAGGCTACCCCCCCGGTA | 1032 | CCTCCCGGCA | 1 | VIC |
| CGCAGGCTACCTCCCCGGTA | 589, 791, **803(AUST-11)** | CCTCTCGGCA | 6 | VIC [3], WA [3] |
| CGCAGGCTACCTCCCGAGTG | 20 | CCTCTCGGCG | 3 | NSW [2], WA [1] |
| CGCAGGCTACCTCCTGGGTG | **508(AUST-11)**, 937 | CCTCTCGGCG | 1 | QLD |
| CGCAGGCTATCCCCTGGGTA | 1,101,199 | CCTCCCGATA | 1 | QLD |
| CGCAGGCTATCCTTCCGGTA ** | NT5 | CCTCCCGGTA | 1 | QLD |
|  |  | TCTCCCGGTA | 1 | VIC |
| CGCAGGCTCCCCCCTGGGCG | NT6 | CCTCCCAGTG | 1 | NSW |
| CGCAGGCTCCCCTCCCGGTA | 385 | CCTCCCGGCA | 2 | NSW |
| CGCAGGCTCCCCTCCCGGTG | 443 | CCTCCCGGTG | 1 | QLD |
| CGCAGGGCCCCCTTCGGGCG | **782(AUST-08), 783(AUST-08), 784(AUST-08), 785(AUST-08)** | CCTCCCAGTG | 13 | NSW [3], SA [10] |
| CGCCGGCCCCCCTTCGGGCG | 378, 638, 797 | CCCCCCAGTG | 1 | QLD |
| CGCCGGCTACCTCCCCGGTA | NT7 | CCCCTCGGCA | 1 | VIC |
| CGCCGGCTACCTTTCCGGCA | 1,703,673,739,971,310 | CCCCTCGGCA | 2 | QLD [1], WA [1] |
| CGCCGGCTATCCCCCCAGTA | 494 | CCCCCCGGCA | 1 | QLD |
| CGCCGGCTATCCCCTGAGTG | 796 | CCCCCCGGCG | 2 | QLD [1], VIC [1] |
| CGCCGGCTCCCCCCCCGACA ** | **179(AUST-10,-12,-14,-26)**,180,353 | CCCCCCAGTA | 7 | NSW [2], QLD [1], VIC [2], WA [2] |
|  |  | CCTCCCAGTA | 1 | VIC |
| CGCCGGCTCCCTTCTGGGTA | 1079 | CCTCTCGGTA | 1 | NSW |
| CGCCGGGCCTCCTCTGAGTG | **809(AUST-22)** | CCCCCCGGCG | 1 | QLD |
| CGTAAGCTATCCCCCCGGTG | 189 | CTCTCCGGCG | 1 | VIC |
| CGTCGACTACCTCCCCGGTA ** | **649(AUST-01)** | CTCCTCGGCA **(AUST-01)** | 80 | NSW [19], QLD [9], SA [14], VIC [33], WA [5] |
|  |  | CTTCTCGGCA^2^ | 1 | NSW |
| CGTCGGCCACTCTTCCGGCG | **655(AUST-20)**, 709 | CTCCCCAGTG | 1 | VIC |
| CGTCGGCCATCCCCCCGGTA | NT8 | CTCCCCGGTA | 1 | NSW |
| CGTCGGCCCTCCCCCCGACA | NT9 | CTCCCCAGCA | 1 | VIC |
| CGTCGGCCCTCCCCTGGGTG | 856 | CTCCCCGGCG | 1 | QLD |
| CGTCGGCTACCCCCCGAGTA | 226, 598, 847, 896, 1087, 1237 | CTCCCTGGCA | 1 | NSW |
| CGTCGGCTACCTCCCGGGCA | NT10 | CTCCTCAGTA | 1 | QLD |
| CGTCGGCTACCTCCTGAGTG | 1189 | CTCCTCGGTG | 1 | QLD |
| CGTCGGCTACCTTCCCGGTA | 653, 934 | CTCCTCGGTA | 1 | VIC |
| CGTCGGCTACCTTCTGGGCG | 859 | CTCCTCAGTG | 3 | VIC [2], WA [1] |
| CGTCGGCTACCTTTTGAGTA | 198, 1048 | CTCCTTGGCA | 1 | NSW |
| CGTCGGCTACCTTTTGAGTG | 529, 569, 857 | CTCCTTGGCG | 4 | QLD [1], SA [1], WA [2] |
| CGTCGGCTATCCCCCCGGTG | 802 | CTCCCCGGCG | 3 | NSW [1], VIC [2] |
| CGTCGGCTATCCCCTGGGCA ** | 398, 399, 401, **810(AUST-17)** | CTCCCCAGTA | 2 | QLD [1], VIC [1] |
|  |  | CTCCCTGGCA | 1 | NSW |
| CGTCGGCTATCCCCTGGGTA | 169, **261(AUST-29)** | CTCCCTGGTA | 1 | VIC |
| CGTCGGCTATCCTTCCGGTA | **17(AUST-15 & Clone C)**, 318, 322, 380, 636, 688, 845, 958, 1255, 1313 | CTCCCCGGTA | 12 | NSW [3], QLD [2], SA [2], VIC [3], WA [2] |
| CGTCGGCTATCCTTCCGGTG | 343, 381, 1256 | CTCCCCGGCG | 1 | VIC |
| CGTCGGCTATCCTTTGGGTA | **497(Dutch-2)**, 544, 895, 1317 | CTCCCTGGCA | 2 | NSW |
| CGTCGGCTCCCCTCCCGGTA | NT11 | CTCCCCGGTA | 1 | SA |
| CGTCGGCTCCCCTCTGAGCA | NT12 | CTCCCTGGCA | 1 | VIC |
| CGTCGGCTCCCCTTCCGGTA | 557 | CTCCCCGGTA | 3 | SA |
| CGTCGGCTCCCCTTCCGGTG | 570, 1228 | CTCCCCGGCG | 2 | SA [1], WA [1] |
| CGTCGGCTCCCTCCTGAGTG ** | 499 | CTCCTCGGCG | 1 | WA |
|  |  | CTTCTCGGCG | 1 | WA |
| CGTCGGCTCCTCTCCCGGTA | 275 | CTCCCCGGTA | 2 | QLD |
| CGTCGGCTCTCCCCCCGGTG | 583, 850 | CTCCCCGGTG | 1 | SA |
| CGTCGGCTCTCCCCTGGACA | **1394(PACS2)** | CTCCCCAGTA | 1 | QLD |
| CGTCGGCTCTCCTCTGGGTA | 483 | CTCCCCGATA | 1 | NSW |
| CGTCGGCTCTCCTTTGGGTA | 147 | CTCCCCGGTA | 2 | NSW |
| CGTCGGCTCTCCTTTGGGTG | 646 | CTCCCCGGTG | 1 | NSW |
| CGTCGGGCACCTTCCCGGTA | 412 | CTCCTCGGTA | 1 | VIC |
| CGTCGGGCACTCCCCCGACA | 851 | CTCCCCAGTA | 1 | QLD |
| CGTCGGGCCTCCTCTGAGTG | 909 | CTCCCCGGCG | 1 | WA |
| TACCAGGCCCCCTCCGAGTG | 89, 307, **308(AUST-24)**, 662, 1028 | TCCCCCGGCG | 1 | WA |
| TACCAGGCCCTCCCCGAGTG | 61, 223, 309, 311, 316, 325, 361, 383, 458, 1251, 1310 | TCCCCCGGCG | 2 | SA [1], WA [1] |
| TATCGGCCCCTCTCCGAGTG | 1259, 1260, 1261, 1262, 1334 | TTCCCCGGCG | 1 | NSW |
| TATCGGCTCCTCTCCGGGCA | 821 | TTCCCCGGCA | 2 | NSW [1], VIC [1] |
| TATCGGGCCCCCCCCGAGCG | 319, 1160, 1400 | TTCCCCGGCG | 2 | SA [1], WA [1] |
| TATCGGGCCCCCCCCGAGTG | 296, 306, 310, 694, 1197 | TTCCCCGGCG | 1 | SA |
| TATCGGGCCCCCTCCGAGTG ** | 65, 107, 109, **253(PA14)**, 297, 338, 342, 377, 532, 773, 815, 923, 1110, 1363 | TTCCCCGGCG | 1 | QLD |
|  |  | TTTCCCGGCG | 1 | WA |
| TATCGGGCCCTCCCCGGGTG | 279, 312, 1020, 1071, 1253 | TTTCCCGGCG | 1 | WA |
| TATCGGGCCCTCTCCGAGTG | 63, 315, 701, 759, 816, 829, 926, 1093, 1138, 1146, 1254 | TTCCCCGGCG | 4 | NSW [2], WA [2] |
| TGCAAGCTACCCCCTGGACA | 13, **155(AUST-10,-14,-19,-37)**, 280, 541, 579, 677, **786(AUST-19)**, 1276, 1316, 1335 | TCTTCCAGTA | 8 | QLD [2], SA [1], VIC [2], WA [3] |
| TGCAAGCTACCTCCCGAGTA | 114 | TCTTTTGGTA | 1 | WA |
| TGCAAGCTACCTCCTGGACG | 14 | TCTTTCAGTG | 1 | WA |
| TGCAAGCTACCTCTTGGGTG | 128, 450, 676 | TCTTTCGGCG | 1 | WA |
| TGCAAGCTACCTTCCCGGTA | **775(AUST-02)** | TCTTTCGGTA **(AUST-02)** | 48 | NSW [3], QLD [26], SA [1], WA [18] |
|  |  | TCTTTCGGVA, incomplete | 1 | QLD |
| TGCAAGCTACCTTCCGGGTA | 807 | TCTTTCGATA | 2 | QLD [1], WA [1] |
| TGCAAGCTACCTTCTGGGTA | 778 | TCTTTCGGTA **(AUST-02)** | 1 | QLD |
| TGCAAGCTACCTTTCCGGCG | 151 | TCCTTCAGTG | 2 | NSW [1], VIC 1] |
| TGCAAGCTACCTTTTGGACA | 11 | TCTTTCAGTA | 1 | QLD |
| TGCAAGCTATCCCCCCGGTG | **12(AUST-33)** | TCTTCCGGTG | 1 | QLD |
| TGCAAGCTATCCTCCCAGCG | **236(AUST-32)**, 239, 240 | TCTTCCGGTG | 2 | SA |
| TGCAAGCTATCCTTTGGACG | 1040 | TCTTCCAGCG | 2 | NSW |
| TGCAAGCTCCCCCTTGGGCG | NT13 | TCTTCCAGTG | 5 | VIC |
| TGCAAGCTCCCCTCCGAGTG | 793 | TCCTCCGGCG | 1 | WA |
| TGCAAGCTCCCCTTTCAGCG | 16 | TCTTCCGGCG | 1 | VIC |
| TGCAAGCTCCCCTTTGGGCG ** | **242(AUST-03)**, 996 | CCTTCCAGTG | 1 | VIC |
|  |  | TCTTCCAGTG | 9 | NSW [1], SA [1], VIC [6], WA [1] |
| TGCAAGCTCTCCCCCCGGTG | 1397 | TCTTCCGGCG | 1 | SA |
| TGCAAGGCCCCCTCCGGGTG | 644, 935 | TCTTCCGGTG | 1 | QLD |
| TGCAAGGCCCCCTTTGGGTG | 1014 | TCCTCCGGTG | 1 | QLD |
| TGCAAGTTACCCTCCCGGTA | NT14 | TCTTCCGGCA | 1 | WA |
| TGCAGGCTCCCTCCCCAGCA | 699 | TCTCTCGGCA | 2 | VIC [1], WA [1] |
| TGCCAGGCCCTCTCCCAGTG | 352 | TCCCCCGGCG | 1 | VIC |
| TGCCGGCTACCCCCCCAGCG | 876 | TCCCCCAGCG | 1 | WA |
| TGCCGGCTACCTCCTGAGTA | 266 | TCCCTCGGCA | 3 | NSW [1], QLD [1], SA [1] |
| TGCCGGCTACCTTTCCAGTA | NT15 | TCCCTCGGTA | 1 | QLD |
| TGCCGGCTATCCCCCCGACA ** | 787, **788(AUST-04)** | TCCCCCAGTA | 16 | NSW [1], QLD [1], SA [1], VIC [11], WA [2] |
|  |  | TCTCCCAGTA | 1 | NSW |
| TGCCGGCTATCCCCCCGGCA | **822(AUST-11)**, **1239(M18)** | TCCCCCAGTA | 2 | QLD [1], SA [1] |
| TGCCGGCTCCCCCCTGAGCA | 1148, 1396 | TCCCCCGGCA | 1 | SA |
| TGCCGGCTCCCCCCTGGGCA | 554, **804(AUST-11)** | TCCCCCAGTA | 1 | VIC |
| TGCCGGCTCTCCTCTGGGTG | NT16 | TCCCCTGGTG | 1 | NSW |
| TGCCGGGCATCCTCCGAATG | 871 | TCCCCCGGCG | 1 | VIC |
| TGCCGGGCATCCTCCGGACG | 870 | TCCCCCAGTG | 2 | NSW [1], QLD [1] |
| TGCCGGGTCTCCTTTGGACA | 285 | TCCCCCGGCA | 3 | VIC [2], WA [1] |
| TGTCGGCCATCCCCCCGGTA | 2 | TTCCCCGGTA | 1 | QLD |
| TGTCGGCCCCTCTCCCAGCG | NT17 | TTCCCCAGTG | 1 | VIC |
| TGTCGGCTACCCTTCCGACA | 260, 264, 503 | TTCCCCAGTA | 3 | QLD [1], WA [2] |
| TGTCGGCTACCCTTCGGGTA | NT18 | TTCCCTGGCA | 1 | QLD |
| TGTCGGCTACCTCCCCGGTG ** | **146(LES)**, 374, 467, 681, 683, 970 | TTCCTCAGTG | 1 | QLD |
|  |  | TTCCTCGGTG | 1 | VIC |
| TGTCGGCTACCTCCCGAGTG | 635 | TTCCTCGGTG | 1 | NSW |
| TGTCGGCTACCTCCTGGGCG | 792, 1340 | TTCCTCAGTG | 2 | VIC [1], WA [1] |
| TGTCGGCTACCTCCTGGGTG | NT19 | TTCCTCGGTG | 1 | WA |
| TGTCGGCTACCTTCCCAGTA | 834 | TTCCTCGGTA | 1 | QLD |
| TGTCGGCTACCTTTCCAGCG | 591, 761, 1049 | TTCCTCGGCG | 1 | WA |
| TGTCGGCTACCTTTCCGGTA | NT20 | TTCCTCGGTA | 1 | QLD |
| TGTCGGCTACCTTTCGGGTA ** | 209, 268, **274(AUST-05,-09,-18,-25,&-31)**, 466, 546, **781 (AUST-05)**, 936, **1043(AUST-09)**, 1068, 1089, 1301, 1326 | TTCCTCGACA | 1 | QLD |
|  |  | TTCCTCGATA | 37 | NSW [6], SA [29], VIC [1], WA [1] |
|  |  | TTTCTCGATA | 3 | NSW |
| TGTCGGCTACTCCTCCAGTA | NT21 | TTCCCCGGCA | 1 | SA |
| TGTCGGCTATCCCCCCGGTA | 132, 212, 607, 615, 665 | TTCCCCGGCA | 1 | NSW |
| TGTCGGCTATCCCCTGAGTA | NT22 | TTCCCCGGCA | 1 | VIC |
| TGTCGGCTATCCCCTGGGTG | 731, 838, 1052 | TTCCCTGGTG | 5 | NSW [1], QLD [1], SA [3] |
| TGTCGGCTATCCTCCGGGTG | 112, 395, 841 | TTCCCCGATG | 1 | VIC |
| TGTCGGCTATCCTTTGGGCA | 668 | TTCCCCAGTA | 1 | NSW |
| TGTCGGCTCCCCCCCCAGTG | 1232 | TTCCCCGGCG | 2 | NSW [1], QLD [1] |
| TGTCGGCTCCCCCCTGGGTA | 252, 411, 429, 495, 702, 984 | TTCCCCGGTA | 1 | NSW |
| TGTCGGCTCCCCTCCGGGTG | 48, 795, 1354 | TTCCCCGGTG | 1 | WA |
| TGTCGGCTCCCCTTCCGGTG | 840 | TTCCCCGATG | 2 | VIC |
| TGTCGGCTCCCTTCCGGGTG | 1210 | TTCCTCGGTG | 1 | WA |
| TGTCGGCTCTCCCCCCGACG | 633 | TTCCCCAGTG | 1 | NSW |
| TGTCGGCTCTCCTCTCGGTA | 162 | TTCCCCGGCA | 2 | SA [1], WA [1] |
| TGTCGGCTCTCCTTCCAGTA | 27, 294, 334, 1208, 1324 | TTCCCCGGCA | 2 | QLD [1], VIC [1] |
| TGTCGGGCCCCCTCCGAGTG | 345, 620, 674 | CTCCCCGGCG | 1 | WA |
| TGTCGGGTCCCCCCCGGGCA | NT23 | TTCCCTAGTA | 1 | WA |
| [C/T]G[T/C]CG[A/G]CTA[T/C]C[C/T]CCCCG[G/A][T/C]A* | incomplete | CCCCCCAGTA | **1** | VIC |
| NGNNNNNNNNNN[T/C]NNNGGNG* | incomplete | CCCCCCAGTG | **1** | WA |
| CNCAAGCTATCCCCCCGGCA | incomplete | CCTCCCAGTA | **1** | VIC |
| NGNNNNNNNNNCCNNNAGNN | incomplete | CCTCCCGGCA | **1** | WA |
| CGCAAGCTACCTCCCCAGTN | incomplete | CCTCTCGGCA | **1** | WA |
| CGCAGGCNACCTCCCCGGTA | incomplete | CCTCTCGGCA | **1** | VIC |
| NNNNNNNNNNNNCNNNNNNN | incomplete | CCTCTCGGTA | **1** | NSW |
| NGTCGGCTATCCTTCGGGTA | incomplete | CTCCCCGGCA | **1** | WA |
| NGTCGGGCCTCCTCTGAGTG | incomplete | CTCCCCGGCG | **1** | WA |
| [C/T]GTCG[A/G]CTACCT[T/C][C/T]C[G/C]GGTA* | incomplete | CTCCTCGACA | **1** | NSW |
| NNNNNACNNNNNCNNNGGTA | incomplete | CTCCTCGGCA **(AUST-01)** | **1** | VIC |
| CNTCGACTACCTCCCCGGTA | incomplete | CTCCTCGGCA **(AUST-01)** | **1** | NSW |
| CGNNNNNNNNNTCCNNGGTA | incomplete | CTCCTCGGCA **(AUST-01)** | **1** | SA |
| CGTCGACTACCTCCCCGGTN | incomplete | CTCCTCGGCA **(AUST-01)** | **1** | VIC |
| TGCCNGGTCCCCCCTGGGCG | incomplete | TCCCCCAGTG | **1** | SA |
| TGNNNGCTACCTCCTGAGTA | incomplete | TCCCTCGGCA | **1** | SA |
| TGCAAGCTATCCTTTGGAC[G/A]* | incomplete | TCTTCCAGCG | **1** | NSW |
| TGTCGGCTCCCCTCTGGGNG | incomplete | TTCCCCAGTG | **1** | QLD |
| TGTCGGCTCCTCTTTGGGTN | incomplete | TTCCCCGGCA | **1** | WA |
| TATCGGGCCCTNTCCGGGTG | incomplete | TTCCCCGGCG | **1** | WA |
| TGNNNNNNNTNCCCNCGGNN | incomplete | TTCCCCGGTG | **1** | NSW |
| NNNNNNNNNNNNNNNNNNNN | incomplete | TTCCTCGATA | **1** | NSW |
| NNNNNNNNNNNNCNNNNNNA | incomplete | TTCCTCGATA | **1** | SA |
| TGNNNNNNNNNTTTNNGGNA | incomplete | TTCCTCGATA | **1** | SA |
| TGTCGGCTCCTCTTTGGGTN | incomplete | TTTCCCGGCV, incomplete | **2** | VIC |
| TGTCGGCTACCTTTNGGGTA | incomplete | TVCCTCGATA, incomplete | **1** | SA |
| CNNNNNNNNNNNCNNNNNTN | incomplete | VTCVVVVACV, incomplete | **1** | VIC |
| TATCGNNTNNNNCNCGNNTA | incomplete | VTVVTVVAVV, incomplete | **1** | VIC |
| TGCAAGCTANCTTCCCGGTA | incomplete | VVVVVTAACV, incomplete | **1** | QLD |
| NNNNNNNNNNNNCNNNNNTN | incomplete | VVVVVVGVVV, incomplete | **1** | NSW |
| NNNNNNNNNNCTCNNNNNTN | incomplete | VVVVVVGVVV, incomplete | **1** | NSW |
| NNTNNNNNNNNNCNNNAGTN | incomplete | VVVVVVGVVV, incomplete | **1** | NSW |
| NNNNNNNNNNCTNNNNNNN | incomplete | VVVVVVVACV, incomplete | **1** | VIC |
| NNNNNNNNNNNNCNNNNNCN | incomplete | VVVVVVVVVV, incomplete | **1** | NSW |

** These 11 iPLEX20SNP profiles were further discriminated by the HRM10SNPassay; N = no SNP, SNP not called by iPLEX; V = variation, SNP not called by HRM; incomplete = incomplete SNP profiles due to one or more SNPs not called by the iPLEX or HRM methods, or because of mixed calls for some SNPs in the iPLEX*; ^1^This isolate provided a HRM10SNP profile consistent with AUST-06 upon repeat testing; ^2^This isolate provided a HRM10SNP profile consistent with AUST-01 upon repeat testing.
