# Supplementary material for: A comparison of two informative SNP-based strategies for typing Pseudomonas aeruginosa isolates from patients with cystic fibrosis
Source: BMC Infect Dis. 2014 Jun 5;14:307. doi: 10.1186/1471-2334-14-307 (PMC4053291; doi:10.1186/1471-2334-14-307)
Supplement: Additional file 4: Table S4 — HRM10SNP profiles further discriminated by the iPLEX20SNP assay. iPLEX20SNP predicted STs or HRM10SNP profiles consistent with recognised P. aeruginosa strains are indicated in parentheses. [file 1471-2334-14-307-S4.docx]

**Table S4.** HRM10SNP profiles further discriminated by the iPLEX20SNP assay. iPLEX20SNP predicted STs or HRM10SNP profiles consistent with recognised *P. aeruginosa* strains are indicated in parentheses.

| HRM10SNP | iPLEX20SNP | iPLEX20SNP predicted MLST | no. |
| --- | --- | --- | --- |
| CCCCCCGGCG | CGCAGGCCCCTCCCCGGGTG | 195, 224, 349, 359, 719, 961, 977, 1077, 1183, 1221, 1222, 1266, 1278 | 1 |
|  | CGCCGGCTATCCCCTGAGTG | 796 | 2 |
|  | CGCCGGGCCTCCTCTGAGTG | **809(AUST-22)** | 1 |
| CCCCTCGGCA | CGCCGGCTACCTCCCCGGTA | NT | 1 |
|  | CGCCGGCTACCTTTCCGGCA | 170, 367, 373, 997, 1315 | 2 |
| CCTCCCAGTA | CGCAAGCCCCTCTTTGGGCA | 740 | 1 |
|  | CGCAAGCTATCCCCCCGGCA | **262(AUST-07)**, 774, 1165 | 4 |
|  | CGCCGGCTCCCCCCCCGACA | **179(AUST-10,-12,-14,&-26)**,180,353 | 1 |
| CCTCCCAGTG | CGCAAGCTACTCTTCCGGCG | 902 | 1 |
|  | CGCAAGCTATCCCCTGGACG | 962 | 1 |
|  | CGCAAGGTCCCCTTTGAGCG | 794 | 2 |
|  | CGCAGGCCCCCCTTCGGGCG | NT | 1 |
|  | CGCAGGCTCCCCCCTGGGCG | NT | 1 |
|  | CGCAGGGCCCCCTTCGGGCG | **782(AUST-08), 783(AUST-08), 784(AUST-08), 785(AUST-08)** | 13 |
| CCTCCCGGCA | CGCAGGCTACCCCCCCGGTA | 1032 | 1 |
|  | CGCAGGCTCCCCTCCCGGTA | 385 | 2 |
| CCTCCCGGTA | CGCAAGCTATCCTTCGGGTA | NT | 1 |
|  | CGCAAGCTCCTCTTTCGGTA | **277(AUST-36)**, 364, 1128, 1390 | 1 |
|  | CGCAGGCTATCCTTCCGGTA | NT | 1 |
| CCTCCCGGTG | CGCAAGGTCCCCCCCGGGTG | **389(AUST-13)** | 2 |
|  | CGCAGGCTCCCCTCCCGGTG | 443 | 1 |
| CCTCTCAGTG | CGCAAGCTACCTCCTGGACG | NT | 1 |
|  | CGCAAGGTACCTCCTGGGCG | **800(AUST-13)** | 2 |
| CCTCTCGGCA | CGCAAGCTACCTCCCCAGTA | **882(AUST-11)**, 1151, 1233 | 2 |
|  | CGCAGGCTACCTCCCCGGTA | 589, 791, **803(AUST-11)** | 6 |
| CCTCTCGGCG | CGCAAGTTCTCTTCTGAGTG | 697 | 3 |
|  | CGCAGGCTACCTCCCGAGTG | 20 | 3 |
|  | CGCAGGCTACCTCCTGGGTG | **508(AUST-11)**, 937 | 1 |
| CCTCTCGGTA | CGCAAGCTACCTCCCCGGTA | 384, **1037(AUST-11)** | 1 |
|  | CGCAAGCTACCTTCCCGGTA | 232, **241(AUST-28)**, 247, 379, 471, 577 | 4 |
|  | CGCCGGCTCCCTTCTGGGTA | 1079 | 1 |
| CTCCCCAGTA | CGTCGGCTATCCCCTGGGCA | 398, 399, 401, **810(AUST-17)** | 2 |
|  | CGTCGGCTCTCCCCTGGACA | **1394(PACS2)** | 1 |
|  | CGTCGGGCACTCCCCCGACA | 851 | 1 |
| CTCCCCGGCG | CGTCGGCCCTCCCCTGGGTG | 856 | 1 |
|  | CGTCGGCTATCCCCCCGGTG | 802 | 3 |
|  | CGTCGGCTATCCTTCCGGTG | 343, 381, 1256 | 1 |
|  | CGTCGGCTCCCCTTCCGGTG | 570, 1228 | 2 |
|  | CGTCGGGCCTCCTCTGAGTG | 909 | 1 |
|  | TGTCGGGCCCCCTCCGAGTG | 345, 620, 674 | 1 |
| CTCCCCGGTA | CGTCGGCCATCCCCCCGGTA | NT | 1 |
|  | CGTCGGCTATCCTTCCGGTA | **17(AUST-15 & Clone C)**, 318, 322, 380, 636, 688, 845, 958, 1255, 1313 | 12 |
|  | CGTCGGCTCCCCTCCCGGTA | NT | 1 |
|  | CGTCGGCTCCCCTTCCGGTA | 557 | 3 |
|  | CGTCGGCTCCTCTCCCGGTA | 275 | 2 |
|  | CGTCGGCTCTCCTTTGGGTA | 147 | 2 |
| CTCCCCGGTG | CGTCGGCTCTCCCCCCGGTG | 583, 850 | 1 |
|  | CGTCGGCTCTCCTTTGGGTG | 646 | 1 |
| CTCCCTGGCA | CGTCGGCTACCCCCCGAGTA | 226, 598, 847, 896, 1087, 1237 | 1 |
|  | CGTCGGCTATCCCCTGGGCA | 398, 399, 401, **810(AUST-17)** | 1 |
|  | CGTCGGCTATCCTTTGGGTA | **497(Dutch-2)**, 544, 895, 1317 | 2 |
|  | CGTCGGCTCCCCTCTGAGCA | NT | 1 |
| CTCCTCGGTA | CGTCGGCTACCTTCCCGGTA | 653, 934 | 1 |
|  | CGTCGGGCACCTTCCCGGTA | 412 | 1 |
| TCCCCCAGTA | TGCCGGCTATCCCCCCGACA | 787, **788(AUST-04)** | 16 |
|  | TGCCGGCTATCCCCCCGGCA | **822(AUST-11)**, **1239(M18)** | 2 |
|  | TGCCGGCTCCCCCCTGGGCA | 554, **804(AUST-11)** | 1 |
| TCCCCCGGCA | TGCCGGCTCCCCCCTGAGCA | 1148, 1396 | 1 |
|  | TGCCGGGTCTCCTTTGGACA | 285 | 3 |
| TCCCCCGGCG | TACCAGGCCCCCTCCGAGTG | 89, 307, **308(AUST-24)**, 662, 1028 | 1 |
|  | TACCAGGCCCTCCCCGAGTG | 61, 223, 309, 311, 316, 325, 361, 383, 458, 1251, 1310 | 2 |
|  | TGCCAGGCCCTCTCCCAGTG | 352 | 1 |
|  | TGCCGGGCATCCTCCGAATG | 871 | 1 |
| TCTTCCAGTG | TGCAAGCTCCCCCTTGGGCG | NT | 5 |
|  | TGCAAGCTCCCCTTTGGGCG | **242(AUST-03)**, 996 | 9 |
| TCTTCCGGCG | TGCAAGCTCCCCTTTCAGCG | 16 | 1 |
|  | TGCAAGCTCTCCCCCCGGTG | 1397 | 1 |
| TCTTCCGGTG | TGCAAGCTATCCCCCCGGTG | **12(AUST-33)** | 1 |
|  | TGCAAGCTATCCTCCCAGCG | **236(AUST-32)**, 239, 240 | 2 |
|  | TGCAAGGCCCCCTCCGGGTG | 644, 935 | 1 |
| **TCTTTCGGTA**  **(AUST-02)** | TGCAAGCTACCTTCCCGGTA | **775(AUST-02)** | 48 |
|  | TGCAAGCTACCTTCTGGGTA | 778 | 1 |
| TTCCCCAGTA | TGTCGGCTACCCTTCCGACA | 260, 264, 503 | 3 |
|  | TGTCGGCTATCCTTTGGGCA | 668 | 1 |
| TTCCCCAGTG | TGTCGGCCCCTCTCCCAGCG | NT | 1 |
|  | TGTCGGCTCTCCCCCCGACG | 633 | 1 |
| TTCCCCGATG | TGTCGGCTATCCTCCGGGTG | 112, 395, 841 | 1 |
|  | TGTCGGCTCCCCTTCCGGTG | 840 | 2 |
| TTCCCCGGCA | TATCGGCTCCTCTCCGGGCA | 821 | 2 |
|  | TGTCGGCTACTCCTCCAGTA | NT | 1 |
|  | TGTCGGCTATCCCCCCGGTA | 132, 212, 607, 615, 665 | 1 |
|  | TGTCGGCTATCCCCTGAGTA | NT | 1 |
|  | TGTCGGCTCTCCTCTCGGTA | 162 | 2 |
|  | TGTCGGCTCTCCTTCCAGTA | 27, 294, 334, 1208, 1324 | 2 |
| TTCCCCGGCG | TATCGGCCCCTCTCCGAGTG | 1259, 1260, 1261, 1262, 1334 | 1 |
|  | TATCGGGCCCCCCCCGAGCG | 319, 1160, 1400 | 2 |
|  | TATCGGGCCCCCCCCGAGTG | 296, 306, 310, 694, 1197 | 1 |
|  | TATCGGGCCCCCTCCGAGTG | 65, 107, 109, **253(PA14)**, 297, 338, 342, 377, 532, 773, 815, 923, 1110, 1363 | 1 |
|  | TATCGGGCCCTCTCCGAGTG | 63, 315, 701, 759, 816, 829, 926, 1093, 1138, 1146, 1254 | 4 |
|  | TGTCGGCTCCCCCCCCAGTG | 1232 | 2 |
| TTCCCCGGTA | TGTCGGCCATCCCCCCGGTA | 2 | 1 |
|  | TGTCGGCTCCCCCCTGGGTA | 252, 411, 429, 495, 702, 984 | 1 |
| TTCCTCAGTG | TGTCGGCTACCTCCCCGGTG | **146(LES)**, 374, 467, 681, 683, 970 | 1 |
|  | TGTCGGCTACCTCCTGGGCG | 792, 1340 | 2 |
| TTCCTCGGTA | TGTCGGCTACCTTCCCAGTA | 834 | 1 |
|  | TGTCGGCTACCTTTCCGGTA | NT | 1 |
| TTCCTCGGTG | TGTCGGCTACCTCCCCGGTG | 146, 374, 467, 681, 683, 970 | 1 |
|  | TGTCGGCTACCTCCCGAGTG | 635 | 1 |
|  | TGTCGGCTACCTCCTGGGTG | NT | 1 |
|  | TGTCGGCTCCCTTCCGGGTG | 1210 | 1 |
| TTTCCCGGCG | TATCGGGCCCCCTCCGAGTG | 65, 107, 109, 253, 297, 338, 342, 377, 532, 773, 815, 923, 1110, 1363 | 1 |
|  | TATCGGGCCCTCCCCGGGTG | 279, 312, 1020, 1071, 1253 | 1 |
| **Total** | | | **247** |
